# The efficiency and safety of methimazole and propylthiouracil in hyperthyroidism: A meta-analysis of randomized controlled trials

**DOI:** 10.1097/MD.0000000000026707

**Published:** 2021-07-30

**Authors:** Shuang Tan, Long Chen, Likun Jin, Xiaomin Fu

**Affiliations:** a Department of Acupuncture and Moxibustion, Huguo Temple Hospital of Traditional Chinese medicine affiliated to Beijing University of Chinese Medicine, Beijing; b Department of Chinese medicine, Inner Mongolia Qingshuihe Hospital of Chinese and Mongolian medicine, Inner Mongolia; c Department of Orthopaedics, Beijing Fengsheng Special Hospital of Traditional Medical Traumatology and Orthopaedics, Beijing, China.

**Keywords:** hyperthyroidism, meta-analysis, methimazole, propylthiouracil

## Abstract

**Purpose:**

The aim of this study was to evaluate the efficiency and safety of methimazole (MMI) and propylthiouracil (PTU) in the treatment of hyperthyroidism.

**Methods:**

Articles were searched through the PubMed, EMBASE, Cochrane Library, Web of Science, CNKI, Wanfang, and QVIP. The primary outcomes were clinical efficacy and thyroid hormone levels in MMI and PTU groups. The secondary outcomes were liver function indexes and adverse reactions in MMI and PTU groups. Results were expressed as weighted mean difference (WMD) or odds ratio (OR) with 95% confidence intervals (CIs). The Begg test was applied to assess the publication bias.

**Results:**

Totally, 16 randomized controlled trials were retained in this meta-analysis with 973 patients receiving MMI and 933 receiving PTU. The levels of triiodothyronine (T_3_) (WMD = −1.321, 95% CI: −2.271 to −0.372, *P* = .006), thyroxine (T_4_) (WMD = −37.311, 95% CI: −61.012 to −13.610, *P* = .002), Free T3 (FT3) (WMD = −1.388, 95% CI: −2.543 to −0.233, *P* = .019), Free T_4_ (FT_4_) (WMD = −3.613, 95% CI: −5.972 to −1.255, *P* = .003), and the risk of liver function damage (OR = 0.208, 95% CI: 0.146–0.296, *P* < .001) in the MMI group were lower than those in the PTU group. The thyroid-stimulating hormone level (WMD = 0.787, 95% CI: 0.380–1.194, *P* < .001) and the risk of hypothyroidism (OR = 2.738, 95% CI: 1.444–5.193, *P* = .002) were higher in the MMI group than those in the PTU group.

**Conclusions:**

Although MMI might have higher risk of hypothyroidism than PTU, the efficacy of MMI may be better than PTU in patients with hyperthyroidism regarding reducing T_3_, T_4_, FT_3_, and FT_4_ levels, decreasing the risk of liver function damage and increasing the level of thyroid-stimulating hormone.

**Register number:**

osf.io/ds637 (*https://osf.io/search/*).

## 1 Introduction

Hyperthyroidism is one of the most common endocrine diseases that caused by excessive production of thyroid hormones.^[1]^ Excessive thyroid hormones inhibits the production of serum thyroid-stimulating hormone (TSH).^[2]^ The prevalence of hyperthyroidism is reported to be up to 1.3% in iodine sufficient areas.^[3]^ Higher incidence of it was obtained in females than that in males with the female-to-male ratio of about 5 to 10:1.^[4]^ Hyperthyroidism is clinically manifested by goiter, protruding eyeballs and increased basal metabolic rate.^[5]^ Hyperthyroidism progresses rapidly and once diagnosed, treatment must be taken as soon as possible.

Evidences indicated that hyperthyroidism can elevate the risk of multiple comorbidities, such as cardiovascular, pulmonary diseases, and psychiatric diseases.^[6–8]^ The association between hyperthyroidism and excess mortality has been confirmed by several studies.^[9,10]^ Nowadays, anti-thyroid drugs (ATDs) are one of the main methods for the treatment of patients with hyperthyroidism, which can preserve the function of thyroid hormone production and have low possibility of hypothyroidism.^[5]^ Methimazole (MMI) and propylthiouracil (PTU) are 2 most extensively used ATDs for patients with hyperthyroidism.^[11]^ MMI and PTU are effective inhibitors of thyroid iodide peroxidase, which can catalyze the biosynthesis of thyroid hormone from the initial step.^[12]^ MMI exerts its function by inhibiting the peroxidase activity in the thyroid, and then suppressing the synthesis of triiodothyronine (T_3_) and thyroxine (T_4_)^[13]^ PTU has an inhibitory effect on peroxidase and the iodization of tyrosine in thyroid, thereby restrains the synthesis of T_4_. Meanwhile, PTU can interfere with the transformation from T_4_ to T_3,_ which decreases the level of serum Free T_3_ (FT_3_).^[14,15]^

Although MMI and PTU were validated to have effects on treating hyperthyroidism, they might have adverse reactions. Previously, a study has demonstrated that PTU has a high risk of adverse reactions compared with MMI in the treatment of hyperthyroidism.^[16]^ Meanwhile, another study has suggested that PTU and MMI has a similar risk of adverse events during the treatment of hyperthyroidism.^[17]^ These controversial results require additional studies to make it clear about the clinical outcomes of hyperthyroidism patients after the treatment of PTU and MMI. This meta-analysis was performed to better understand the efficacy and safety of PTU and MMI in the treatment of hyperthyroidism.

## 2 Methods

### 2.1 Search strategy

The study was conducted on July 1st, 2020. Articles were searched through the PubMed, EMBASE, Cochrane Library, Web of Science, CNKI, Wanfang, and QVIP. English database was searched on July 9^th^, 2020, whereas Chinese database was searched on July 14^th^, 2020. We included the terms “Hyperthyroidism” OR “Hyperthyroid” OR “Hyperthyroids” OR “Primary Hyperthyroidism” OR “Hyperthyroidism, Primary” AND “Antithyroid Agents” OR “Agents, Antithyroid” OR “Thyroid Antagonists” OR “Antagonists, Thyroid” OR “Antithyroid Drugs” OR “Drugs, Antithyroid” OR “Goitrogens” OR “Antithyroid Effect” OR “Effect, Antithyroid” OR “Antithyroid Effects” OR “Effects, Antithyroid” OR “methylthiouracil” OR “Alkiron” OR “4-Methyl-2-thiouracil” OR “Thimecil” OR “Metacil” OR “propylthiouracil” OR “6-Propyl-2-Thiouracil” OR “6 Propyl 2 Thiouracil” OR “methimazole” OR “1-Methyl-2-mercaptoimidazole” OR “1 Methyl 2 mercaptoimidazole” OR “Merkazolil” OR “Tiamazol” OR “Thiamazole” OR “Thimazol” OR “Mercasolyl” OR “Mercazolyl” OR “Methymazol” OR “Methylmercaptoimidazole” OR “Mercazol” OR “Mercazole” OR “Metisol” OR “Metizol” OR “Tapazole” OR “Tirodril” OR “Strumazol” OR “Thiamazol Henning” OR “Henning, Thiamazol” OR “Thiamazol Hexal” OR “Hexal, Thiamazol” OR “Thyrozol” OR “Favistan” OR “Methizol” OR “carbimazole” OR “Carbimazole Henning” OR “Neo-Thyreostat” OR “Neomercazole” OR “Neo-Mercazole” OR “Neo Tomizol”. The retrieval style in PubMed was Search: ((((((((((carbimazole [Title/Abstract]) OR (Carbimazole Henning [Title/Abstract])) OR (Neo-Thyreostat [Title/Abstract])) OR (Neomercazole [Title/Abstract])) OR (Neo-Mercazole [Title/Abstract])) OR (Neo Tomizol [Title/Abstract])) OR (((((((((((((((((((((((((methimazole [Title/Abstract]) OR (1-Methyl-2-mercaptoimidazole [Title/Abstract])) OR (1 Methyl 2 mercaptoimidazole [Title/Abstract])) OR (Merkazolil [Title/Abstract])) OR (Tiamazol [Title/Abstract])) OR (Thiamazole [Title/Abstract])) OR (Thimazol [Title/Abstract])) OR (Mercasolyl [Title/Abstract])) OR (Mercazolyl [Title/Abstract])) OR (Methymazol [Title/Abstract])) OR (Methylmercaptoimidazole [Title/Abstract])) OR (Mercazol [Title/Abstract])) OR (Mercazole [Title/Abstract])) OR (Metisol [Title/Abstract])) OR (Metizol [Title/Abstract])) OR (Tapazole [Title/Abstract])) OR (Tirodril [Title/Abstract])) OR (Strumazol [Title/Abstract])) OR (Thiamazol Henning [Title/Abstract])) OR (Henning, Thiamazol [Title/Abstract])) OR (Thiamazol Hexal [Title/Abstract])) OR (Hexal, Thiamazol [Title/Abstract])) OR (Thyrozol [Title/Abstract])) OR (Favistan [Title/Abstract])) OR (Methizol [Title/Abstract]))) OR (((propylthiouracil [Title/Abstract]) OR (6-Propyl-2-Thiouracil [Title/Abstract])) OR (6 Propyl 2 Thiouracil [Title/Abstract]))) OR (((((methylthiouracil [Title/Abstract]) OR (Alkiron [Title/Abstract])) OR (4-Methyl-2-thiouracil [Title/Abstract])) OR (Thimecil [Title/Abstract])) OR (Metacil [Title/Abstract]))) OR (((((((((((Antithyroid Agents [Title/Abstract]) OR (Agents, Antithyroid [Title/Abstract])) OR (Thyroid Antagonists [Title/Abstract])) OR (Antagonists, Thyroid [Title/Abstract])) OR (Antithyroid Drugs [Title/Abstract])) OR (Drugs, Antithyroid [Title/Abstract])) OR (Goitrogens [Title/Abstract])) OR (Antithyroid Effect [Title/Abstract])) OR (Effect, Antithyroid [Title/Abstract])) OR (Antithyroid Effects [Title/Abstract])) OR (Effects, Antithyroid [Title/Abstract]))) AND (((((Hyperthyroidism [Title/Abstract]) OR (Hyperthyroid [Title/Abstract])) OR (Hyperthyroids [Title/Abstract])) OR (Primary Hyperthyroidism [Title/Abstract])) OR (Hyperthyroidism, Primary [Title/Abstract])). The retrieved literatures were imported into EndNoteX9, and the literatures after preliminary screening were conducted by reading the title and abstract. Then, the literatures that did not meet the requirements were excluded after reading the full text, and the remaining literature was finally included in this study. Our study was performed according to Preferred Reporting Items for Systematic Reviews and Meta-analysis (PRISMA) guidelines, for the Institutional Review Board's approval or the informed consent are not necessarily required for meta-analysis.

### 2.2 Eligibility criteria

Inclusion criteria were first hyperthyroidism patients. The diagnostic criteria of hyperthyroidism are based on clinical symptoms: metabolic syndromes including heat unbearable, sweat, flustered, hand shake, easy to hunger, hyperphagia, emaciation characterized by goiter, ophthalmic sign, among others; and laboratory examinations: the serum levels of T_3_ and T_4_, FT_3_, free T_4_ (FT_4_) are increased, and the serum level of TSH is decreased; second, experimental group: treated with MMI, control group: treated with PTU; third, randomized controlled trials (RCTs); fourth, English and Chinese literatures.

**Exclusion criteria were:** animal experiments; articles with different study topics with our study; articles impossible to extract data; conference articles, dissertations, case reports, meta-analyses, and reviews.

### 2.3 Methodological quality appraisal

For the RCTs included in this study, the modified Jadad scale was used to evaluate their qualities,^[18]^ which has a total score of 7 with 1 to 3 as low quality and 4 to 7 as high quality (Supplementary Table 1–2). Additionally, the Cochrane Collaboration's tool for assessing risk of bias in RCTs was applied to evaluate the quality of included studies.^[19]^ The tool involved in Random Sequence Generation, Allocation Concealment, Blinding of Participants and Personnel, Blinding of Outcome Assessment, Incomplete Outcome Data Addressed, Free of Selective Reporting, and Free of Other Bias. Each was classified as “Yes,” “No,” or “?.” The results of the quality evaluation of included studies were shown in Supplementary Table 3 and Supplementary Figure 6. Moreover, the Grading of Recommendations, Assessment, Development and Evaluation (GRADE) approach was applied to measure the overall quality of evidence included in our study.^[20]^ Evidence was evaluated through two aspects including Decrease quality of evidence (Study limitation, Indirectness, Inconsistency, Imprecision, and Publication bias) and increased quality of evidence (Large magnitude of effect, Residual confounding, and dose–response gradient). The detailed results were depicted in Supplementary Table 4.

### 2.4 Data collection process

All data were assessed by 2 reviewers (ST and LC) who extracted data including author, year, country, length of study, interventions (MMI or PTU), sex, age, number of study subjects, and outcomes indicators: clinical efficacy (effective rate and drug withdrawal rate); thyroid hormone levels (TSH, T_3_, T_4_, FT_3_, FT_4_, thyrotropin receptor antibody [TRAb] and thyroid peroxidase antibody [TPOAb]); liver function indexes (alanine aminotransferase [ALT], aspartate aminotransferase [AST], and alkaline phosphatase [ALP] levels), and adverse reactions (hypothyroidism, liver function damage, rash, pruritus, and leukopenia) (Table 1). When disagreements existed between the 2 reviewers, a consensus was achieved by consulting a third person (LJ).

**Table 1:** Characteristics of articles involved in this meta-analysis.

Author	Year	Country	Length of study	Groups	Intervention	N	Male/Female	Jadad score	Outcomes
Homsanit et al^[22]^	2001	Thailand	3 mo	MMI	15 mg once/day	35	4/31	5	3, 4, 5, 13
				PTU	150 mg once/day	36	5/31		
He^[21]^	2004	China	3 mo	MMI	15 mg once/day	15	5/10	4	3, 4, 7, 8, 13
				PTU	150 mg once/day	15	4/11		
Nakamura^[23]^	2007	Japan	3 mo	MMI	30 mg once/day	98	25/73	6	14, 15, 17
				PTU	300 mg once/day	81	11/70		
Otsuka^[24]^	2012	Japan	3 mo	MMI	30 mg once/day	144	21/123	4	2, 14, 15
				PTU	300 mg once/day	120	11/109		
Ma^[35]^	2014	China	3 mo	MMI	10 mg, 3 times /day for 30 days; then 10 mg, twice/d for 15 days; then 15 mg, once/day for 45 days	50	24/76	4	5, 6, 7, 8, 9
				PTU	100 mg, 3 times/day for 30 day; then 100 mg, twice/day for 15 days; then 50 mg, 3 times/days for 45 days	50			
Xiang^[29]^	2014	China	2 y	MMI	20 mg, once/day for a month; then 2.5 mg, once/day for 1–2 year	23	8/15	4	10, 14, 15, 17
				PTU	100 mg, 3 times/day for a month; then 50 mg, once/day for 1–2 y	23	6/17		
Wang^[33]^	2015	China	2 y	MMI	10 mg, 3 times/day	60	31/29	3	1, 8, 9, 11, 12, 13, 17
				PTU	100 mg, 3 times/day	60	27/33		
He^[32]^	2016	China	1.5 y	MMI	30 mg/day, 3 times/day and then 5–10 mg/day, 3 times/day	50	23/27	2	3, 4, 5
				PTU	100 mg, 3 times/day; then 5–100 mg, 3 times/day	50	22/28		
Liang^[31]^	2016	China	3 mo	MMI	30 mg/day; then 5–10 mg/day	40	0/40	5	10, 11, 12
				PTU	300mg/d; then 50–100 mg/day	40	0/40		
Wang^[25]^	2016	China	6 mo	MMI	10 mg, 3 times/day	50	19/31	4	1, 5, 6, 7, 11, 12, 13, 14, 15, 17
				PTU	100 mg, 3 times/day	50	17/33		
Bai^[36]^	2017	China	3 mo	MMI	10 mg, 3 times/day for 3 wk; then 10 mg, twice/day for 2 wk; then 10 mg, once/day for 3 mo	45	23/22	2	5, 6, 7, 13, 14, 15, 16, 17
				PTU	100, 3 times/day for 3 wk; then 100 mg, 1–2 times/day for 2 wk; then 50 mg, once/day for 3 mo	45	24/21		
Ma^[26]^	2017	China	3 mo	MMI	30 mg/day; then 5–10 mg/day	128	50/78	3	14
				PTU	300 mg/day; then 50–100 mg/day	128	60/68		
Xu^[28]^	2017	China	3 mo	MMI	10 mg, twice/day for 3 mo	45	15/30	5	5, 6, 7, 10, 11,12, 14
				PTU	100 mg, 3 times/day for 3 mo	45	16/29		
Chen^[30]^	2018	China	1 y	MMI	30 mg, once/day; then 5–10 mg/day for 1 y	60	26/34	4	3, 4, 5, 6, 10, 11,12, 14
				PTU	250 mg/day; 40–90 mg/day for 1 y	60	25/35		
Wu^[34]^	2018	China	1 y	MMI	20–40 mg, once or twice	34	15/19	3	1, 5, 6, 7, 14, 15, 16, 17
				PTU	300 mg; then 150–400 mg	34	14/20		
Yang^[27]^	2019	China	6 mo	MMI	30 mg/day; then 5–10 mg/day	96	34/62	4	
				PTU	300 mg/day; then 50–100 mg/day	96	30/66		5, 6, 7, 10

### 2.5 Objectives

The primary objective was to compare the outcomes of patients receiving MMI or PTU including clinical efficacy (effective rate and drug withdrawal rate) and thyroid hormone levels (T_3_ level, T_4_ level, TSH level, FT_3_ level, FT_4_ level, TRAb level, and TPOAb level). The secondary outcomes were liver function indexes ALP level, ALT level, and AST level) and adverse reactions (hypothyroidism, liver function damage, rash, pruritus, and leukocytopenia). Subgroup analysis was conducted according to length of study, literature quality, and the results of Cochrane bias of risk evaluation.

### 2.6 Statistical analysis

Stata15.1 software (Stata Corporation, College Station, TX) was employed for statistical analysis in this meta-analysis. The weighted mean difference (WMD) was used as the effect index for measurement data while odds ratio (OR) were utilized as the effect index for the enumeration data with respective 95% confidence intervals (CIs). Heterogeneity test was performed for each outcome, and random-effects model analysis was performed when the heterogeneity was high (*I*^2^ ≥50%), otherwise, fixed-effects model analysis was adopted. When the difference was statistically significant and the heterogeneity was high (*I*^2^ ≥50%), the research time and literature quality were subjected to subgroup analysis. Meta-regression analysis was used to explore the source of heterogeneity. Sensitivity analysis was performed for all outcomes through reducing the literature by one and see whether the final conclusion has changed. The Begg test was applied to assess the publication bias. A difference of *P* < .05 was statistically significant.

## 3 Results

### 3.1 Included studies

**Figure 1:**
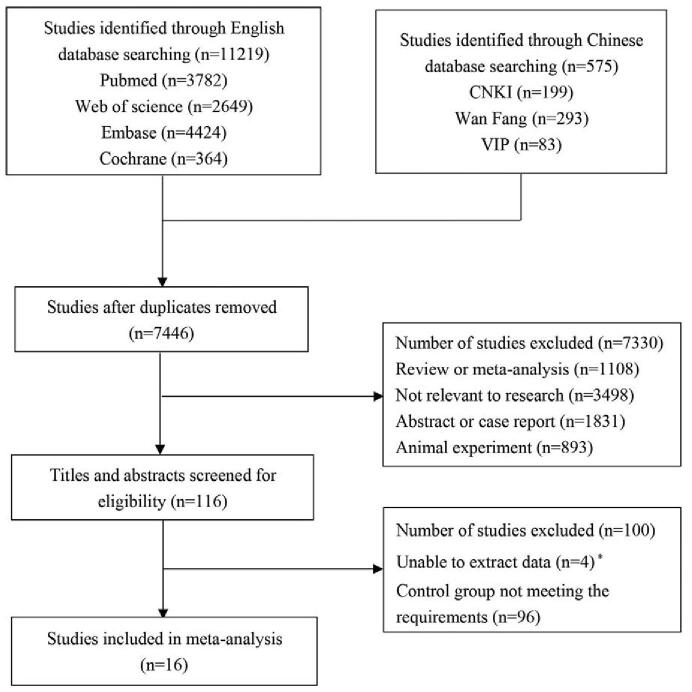
The screen process of the included articles.

According to the search strategy, 11,219 articles were identified through searching English database and 575 articles were identified through retrieving Chinese database. After removing the duplicates, 7446 articles were included. Then 1108 reviews or meta-analysis, 3498 irrelevant researches, 1831 abstracts or case reports, and 893 animal experiments were eliminated. After screening the titles and abstracts, 4 articles unable to extract data and 96 articles with control group not meeting the requirements were excluded. Finally, 16 RCTs were retained.^[21–36]^ In total, 1906 subjects were involved in this study with 973 receiving MMI and 933 receiving PTU. Figure 1 displayed the screen process of the articles.

### 3.2 Overall meta-analysis

The results in this meta-analysis showed that the levels of T_3_ (WMD = −1.321, 95% CI: −2.271 to −0.372, *P* = .006), T_4_ (WMD = −37.311, 95% CI: −61.012 to −13.610, *P* = .002), FT_3_ (WMD = −1.388, 95% CI: −2.543 to −0.233, *P* = .019), FT_4_ (WMD = −3.613, 95% CI: −5.972 to −1.255, *P* = .003), and the risk of liver function damage (OR = 0.208, 95% CI: 0.146–0.296, *P* < .001) in the MMI treatment group were lower than those in the PTU treatment group. The TSH level (WMD = 0.787, 95% CI: 0.380–1.194, *P* < .001) and the risk of hypothyroidism (OR = 2.738, 95% CI: 1.444–5.193, *P* = .002) were higher in the MMI treatment group than those in the PTU treatment group. No significant differences were obtained regarding the effective rate (OR = 0.427, 95% CI: 0.021–8.638), the drug withdrawal rate (OR = 1.135, 95% CI: 0.516–2.498), the levels of TRAb (95% CI: −28.085 to −3.288), TPOAb (WMD = 11.540, 95% CI: −5.873 to −28.952), ALP (WMD = −4.708, 95% CI: −19.606 to −10.189), ALT (WMD = −1.786, 95% CI: −8.078 to −4.506), AST (WMD = −2.149, 95% CI: −10.750 to −6.453), and the risk of rash (OR = 1.419, 95% CI: 0.980–2.056), pruritus (OR = 0.247, 95% CI: 0.099–1.220), leukocytopenia (OR = 0.887, 95% CI: 0.487–1.615) between the MMI treatment group and the PTU treatment group, all *P* > .05 (Table 2).

**Table 2:** Overall data of the meta-analysis.

Outcomes	Indicators	WMD/OR (95% CI)	*P*	*I*^2^
Thyroid hormone levels	T_3_, nmol/L (4)			
	**Overall**	−1.321 (−2.271 to −0.372)	.006	96.4
	Sensitivity	−1.321 (−2.271 to −0.372)		
	Study time			
	3 mo	−2.017 (−2.359 to −1.674)	<.001	0.0
	≥1 y	−0.583 (−1.021 to −0.145)	.009	68.5
	Literature quality			
	High quality	−1.474 (−2.762 to −0.185)	.025	97.5
	Low quality	−0.890 (−1.403 to −0.377)	.001	NA
	Blinding of outcome assessment			
	Yes	−1.474 (−2.762 to −0.185)	.025	97.5
	No	−0.890 (−1.403 to −0.377)	.001	NA
	T_4_, nmol/L (4)			
	**Overall**	−37.311 (−61.012 to −13.610)	.002	98.2
	Sensitivity	−37.311 (−61.012 to −13.610)		
	Study time			
	3 mo	−60.064 (−79.052 to −41.076)	<.001	58.4
	≥1 y	−15.340 (−36.123 to 5.442)	.148	97.8
	Literature quality			
	High quality	−42.640 (−84.080 to −1.199)	.044	98.4
	Low quality	−26.130 (−31.940 to 20.320)	<.001	NA
	Blinding of outcome assessment			
	Yes	−42.640 (−84.080 to −1.199)	.044	98.4
	No	−26.130 (−31.940 to 20.320)	<.001	NA
	TSH, μIU/mL (9)			
	**Overall**	0.787 (0.380–1.194)	<.001	98.0
	Sensitivity	0.787 (0.380–1.194)		
	Study time			
	3 mo	1.385 (−0.374 to 3.145)	.123	98.8
	6 mo	0.105 (−0.107 to 0.316)	.332	65.5
	≥1 y	0.516 (0.284 to 0.747)	<.001	55.4
	Literature quality			
	High quality	0.641 (0.045 to 1.237)	.035	98.1
	Low quality	1.116 (0.233 to 1.999)	.013	96.6
	Blinding of outcome assessment			
	Yes	1.191 (−0.172 to 2.554)	.087	98.8
	No	0.439 (0.132 to 0.746)	.005	94.2
	FT_3_, pmol/L (8)			
	**Overall**	−1.388 (−2.543 to −0.233)	.019	97.7
	Sensitivity	−1.388 (−2.543 to −0.233)		
	Study time			
	3 mo	−1.133 (−3.094 to 0.828)	.258	97.8
	6 mo	−1.532 (−4.609 to 1.545)	.329	99.2
	1 y	−1.767 (−2.992 to −0.542)	.005	92.1
	Literature quality			
	High quality	−1.077 (−2.537 to 0.384)	.149	98.1
	Low quality	−2.311 (−2.667 to −1.955)	<.001	0.0
	Blinding of outcome assessment			
	Yes	−2.791 (−3.351 to −2.230)	<.001	56.3
	No	−0.618 (−1.851 to 0.614)	.326	97.2
	FT_4_, pmol/L (9)			
	**Overall**	−3.613 (−5.972 to −1.255)	.003	98.6
	Sensitivity	−3.613 (−5.972 to −1.255)		
	Study time			
	3 months	−3.254 (−6.664 to 0.156)	.061	98.5
	6 mo	−3.590 (−10.116 to 2.937)	.281	98.3
	1 y	−4.573 (−7.442 to −1.704)	.002	91.2
	Literature quality			
	High quality	−3.979 (−8.071 to 0.114)	.057	98.8
	Low quality	−3.388 (−8.600 to 1.823)	.203	98.0
	Blinding of outcome assessment			
	Yes	−1.807 (−4.280 to 0.0.665)	.152	98.2
	No	−6.759 (−7.448 to −6.071)	<.001	0.0
	TRAb, U/L (3)			
	**Overall**	−12.398 (−28.085 to 3.288)	.121	97.2
	Sensitivity	−12.398 (−28.085 to 3.288)		
	TPOAb, IU/mL (2)			
	**Overall**	11.540 (−5.873 to 28.952)	.194	0.0
	Sensitivity	11.540 (−5.873 to 28.952)		
Liver function indexes	ALP, U/L (4)			
	**Overall**	−4.708 (−19.606 to 10.189)	.536	96.8
	Sensitivity	−4.708 (−19.606 to 10.189)		
	ALT, U/L (4)			
	**Overall**	−1.786 (−8.078 to 4.506)	.578	98.2
	Sensitivity	−1.786 (−8.078 to 4.506)		
	AST, U/L (4)			
	**Overall**	−2.149 (−10.750 to 6.453)	.624	98.4
	Sensitivity	−2.149 (−10.750 to 6.453)		
Clinical efficacy	Effective rate (2)			
	**Overall**	0.427 (0.021 to 8.638)	.579	67.6
	Sensitivity	0.427 (0.021 to 8.638)		
	Drug withdrawal rate (2)			
	**Overall**	1.135 (0.516 to 2.498)	.753	66.8
	Sensitivity	1.135 (0.516 to 2.498)		
Adverse reactions	Hypothyroidism (6)			
	**Overall**	2.738 (1.444 to 5.193)	.002	26.5
	Sensitivity	2.738 (1.444 to 5.193)		
	Liver function damage (9)			
	**Overall**	0.208 (0.146 to 0.296)	<.001	19.3
	Sensitivity	0.208 (0.146 to 0.296)		
	Rash (8)			
	**Overall**	1.419 (0.980 to 2.056)	.064	0.0
	Sensitivity	1.419 (0.980 to 2.056)		
	Pruritus (3)			
	**Overall**	0.247 (0.099 to 1.220)	.099	0.0
	Sensitivity	0.247 (0.099 to 1.220)		
	Leukocytopenia (5)			
	**Overall**	0.887 (0.487 to 1.615)	.696	13.7
	Sensitivity	0.887 (0.487 to 1.615)		
Recurrence	(2)			
	**Overall**	0.420 (0.061 to 2.904)	.379	0.0
	Sensitivity	0.420 (0.061 to 2.904)		

### 3.3 Clinical efficacy

#### 3.3.1 Effective rate

**Figure 2:**
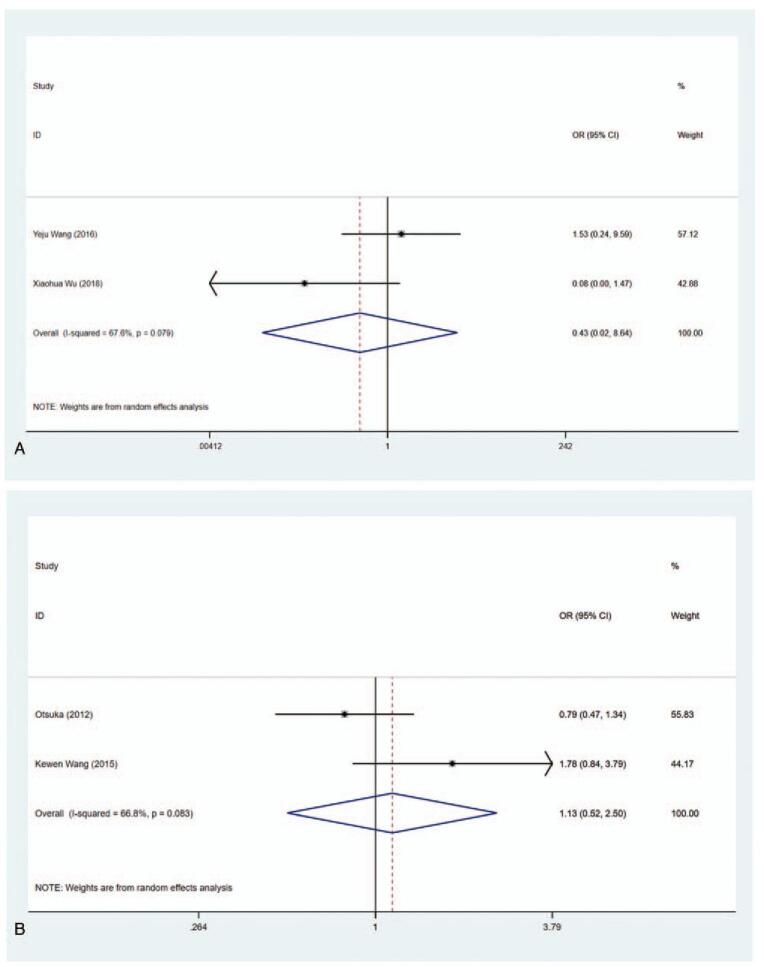
Forest plot for effective rate (A) and drug withdrawal rate (B).

Effective rate = (cured + improved)/total number of cases. Cured means that the symptoms and signs of hyperthyroidism disappear completely and the thyroid hormone level returns to normal. Improved means that the symptoms and signs of hyperthyroidism disappeared, and the serum thyroid hormone level decreased, but still did not return to the normal level. Invalid means that the symptoms and signs of hyperthyroidism repeatedly existed or worsened, and the serum thyroid hormone level never decreased. In total, 2 articles included the data about the effective rate of MMI and PTU. Heterogeneity in the studies showed statistically significant difference (*I*^2^ = 67.6%), so the random-effect model was used for pooled analysis. The results depicted that there was no difference in clinical efficacy between the MMI group and the PTU group (OR = 0.427, 95% CI: 0.021–8.638, *P* = .579) (Fig. 2A, Table 2).

#### 3.3.2 Drug withdrawal rate

The data on drug withdrawal rate were described in 2 articles (*I*^2^ = 66.8%). Similar drug withdrawal rate was obtained in the MMI group and PTU group with no statistical significance (OR = 1.135, 95% CI: 0.516–2.498, *P* = .753) (Fig. 2B, Table 2).

### 3.4 Thyroid hormone levels

#### 3.4.1 T_3_ level, (nmol/L)

Four studies had sufficient data for assessing T_3_ level (nmol/L) in the MMI group and PTU group. The results elucidated that T_3_ level in the MMI treatment group was lower than that of PTU treatment group (WMD = −1.321, 95% CI: −2.271 to −0.372, *P* = .006) (Fig. 3 A, Table 2). The sensitivity analysis showed that WMD = −1.321 (95% CI: −2.271 to −0.372). As substantial heterogeneity was observed in the pooled data (*I*^2^ = 96.4%), subgroup analysis was conducted. According to length of study and literature quality, there were significant differences in 3 months (WMD = −2.017, 95% CI: −2.359 to −1.674, *P* < .001), ≥1 year (WMD = −0.583, 95% CI: −1.021 to −0.145, *P* < .001), high quality (WMD = −1.474, 95% CI: −2.762 to −0.185, *P* = .025), and low quality (WMD = −0.890, 95% CI: −1.403 to −0.377, *P* = .001). (Fig. 3 B and C, Table 2). To explore the sources of heterogeneity, meta-regression was performed concerning length of study (3 months vs ≥1 year) and literature quality (high quality vs low quality). The results demonstrated that length of study and literature quality were not associated with the heterogeneity (*P* > .05).

**Figure 3:**
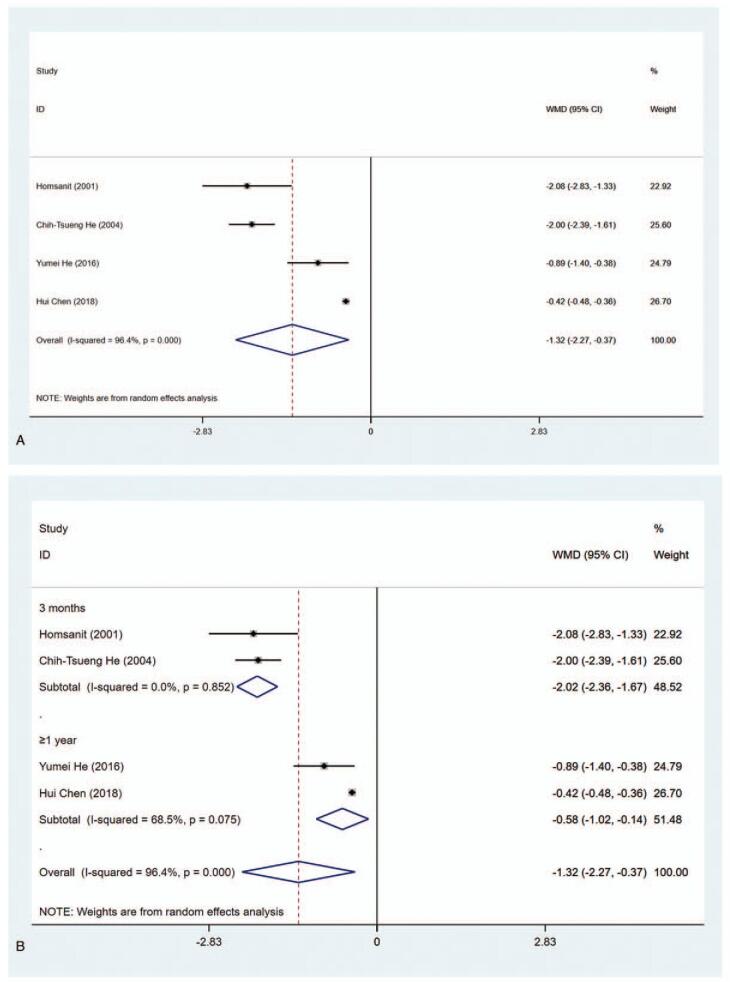
Forest plot for T3 level (A), length of study (B) and literature quality (C).

**Figure 3 (Continued):**
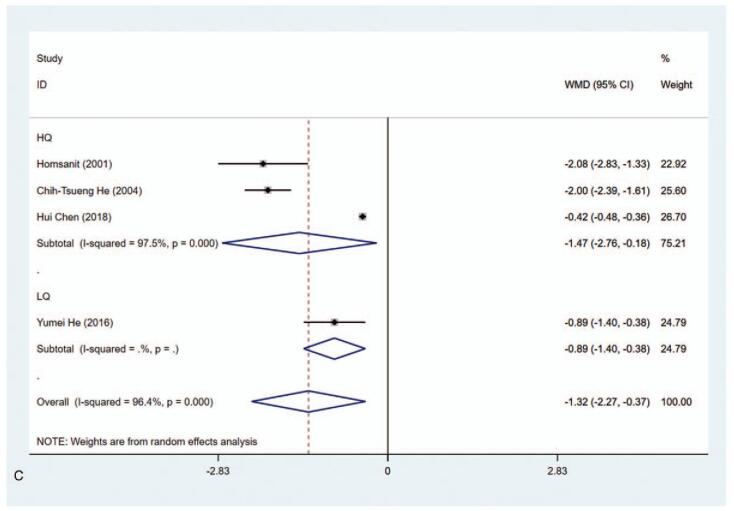
Forest plot for T3 level (A), length of study (B) and literature quality (C).

According to the results of the Cochrane Collaboration's tool for assessing risk of bias in RCTs, 6 studies presented high risk of bias in Blinding of Outcome Assessment. Subgroup analysis was also conducted based on the results of Blinding of Outcome Assessment. The data depicted that there were significant differences in Blinding of Outcome Assessment (Yes) (WMD = −1.474, 95% CI: −2.762 to −0.185, *P* = .025) and Blinding of Outcome Assessment (No) (WMD = −0.890, 95% CI: −1.403 to −0.377, *P* = .001) groups (Supplementary Figure 1, Table 2). The results suggested that T_3_ level in the MMI treatment group was lower than that of PTU treatment group.

#### 3.4.2 T_4_ level (nmol/L)

The data about the level of T_4_ (nmol/L) have been reported in 4 articles. The pooled analysis of data revealed that the level of T_4_ in the MMI treatment group was lower than that in the PTU treatment group (WMD = −37.311, 95% CI: −61.012 to −13.610, *P* = .002) (Fig. 4 A, Table 2). The sensitivity analysis showed that WMD = −37.311 (95% CI: −61.012 to −13.610). Subgroup analysis was conducted in regarding with length of study and literature quality due to the substantial heterogeneity (*I*^2^ = 98.2%). As shown in Figure 4 B and C and Table 2, significant differences were observed in 3 months (WMD = −60.064, 95% CI: −79.052 to −41.076, *P* < .001), high quality (WMD = −42.640, 95% CI: −84.080 to −1.199, *P* = .044) and low quality (WMD = −26.130, 95% CI: −31.940 to −20.320, *P* < .001). Meta-regression was conducted on length of study (3 months vs ≥1 year) and literature quality (high quality vs low quality), showing that length of study and literature quality had no relevant to the heterogeneity (*P* > .05). In addition, significant differences were also seen in Blinding of Outcome Assessment (Yes) (WMD = −42.640, 95% CI: −84.080 to −1.199, *P* = .044) and Blinding of Outcome Assessment (No) (WMD = −26.130, 95% CI: −31.940 to −20.320, *P* < .001) groups (Supplementary Figure 2, Table 2), indicating that T_4_ level in the MMI treatment group was lower than that of PTU treatment group.

**Figure 4:**
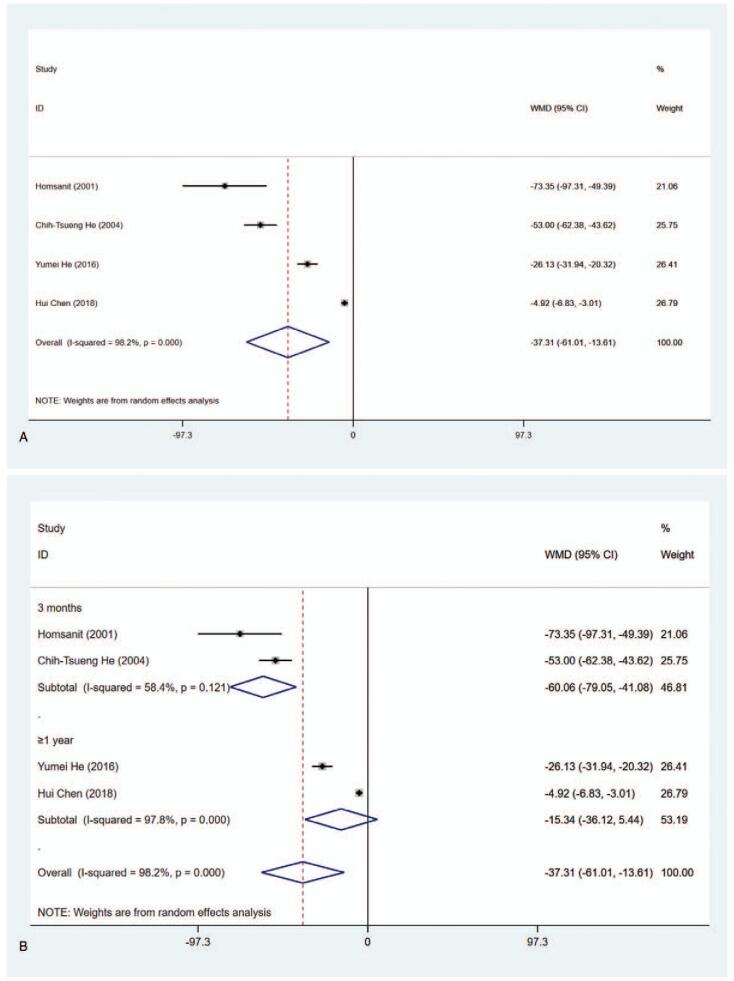
Forest plot for T4 level (A), length of study (B) and literature quality (C).

**Figure 4 (Continued):**
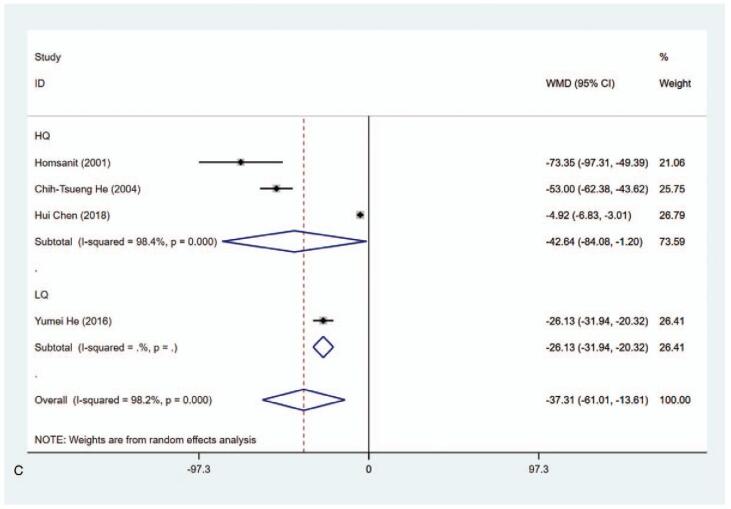
Forest plot for T4 level (A), length of study (B) and literature quality (C).

#### 3.4.3 TSH level (μIU/mL)

The data on the level of TSH (μIU/mL) were available in 9 studies. According to the results of the pooled data analysis, the TSH level was higher in the MMI treatment group than that in the PTU treatment group (WMD = 0.787, 95% CI: 0.380–1.194, *P* < .001) (Fig. 5 A, Table 2). The sensitivity analysis showed that WMD = 0.787 (95% CI: 0.380–1.194). The heterogeneity test results showed statistically significant difference (*I*^2^ = 98.0%). Subgroup analysis indicated the differences were statistically significant in ≥1 year (WMD = 0.516, 95% CI: 0.284–0.747, *P* < .001), high quality (WMD = 0.641, 95% CI: 0.045–1.237, *P* = .035), and low quality (WMD = 1.116, 95% CI: 0.233–1.999, *P* = .013) (Fig. 5 B and C, Table 2). The results of meta-regression analysis on length of study (3 vs 6 months or 3 months vs ≥1 year) and literature quality (high quality vs low quality) disclosed that length of study and literature quality were not the influencing factors of the heterogeneity (*P* > .05). Besides, subgroup analysis in risk of bias concerning Blinding of Outcome Assessment showed evident difference in Blinding of Outcome Assessment (No) group (WMD = 0.439, 95% CI: 0.132–0.746, *P* = .005) (Supplementary Figure 3, Table 2), implying that T_3_ level in the MMI treatment group was lower than that of PTU treatment group in studies with risk of bias in Blinding of Outcome Assessment.

**Figure 5:**
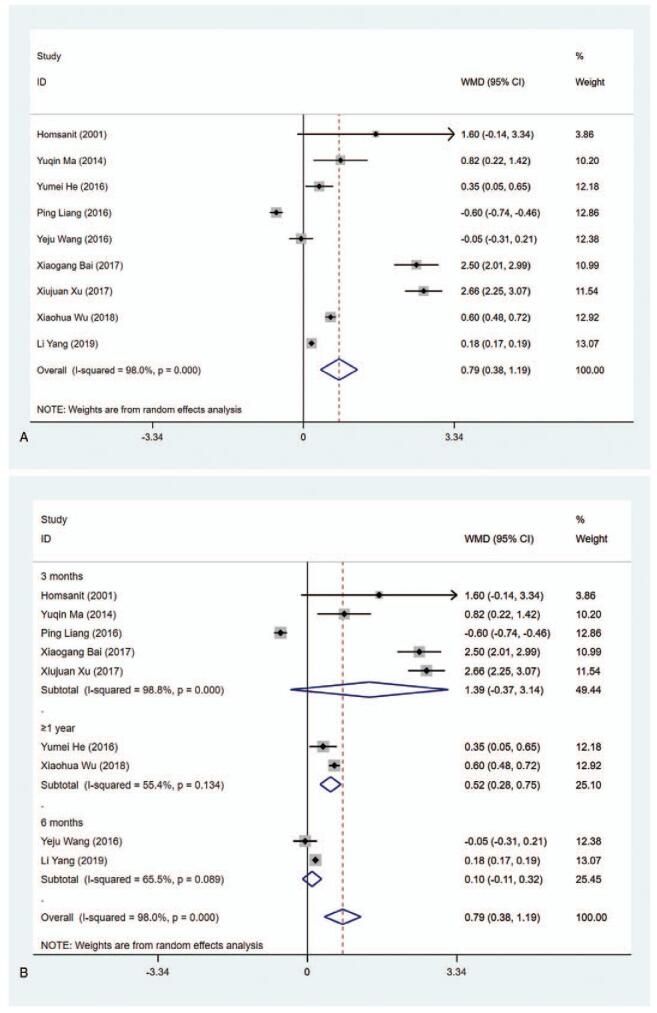
Forest plot for TSH level (A), length of study (B) and literature quality (C).

**Figure 5 (Continued):**
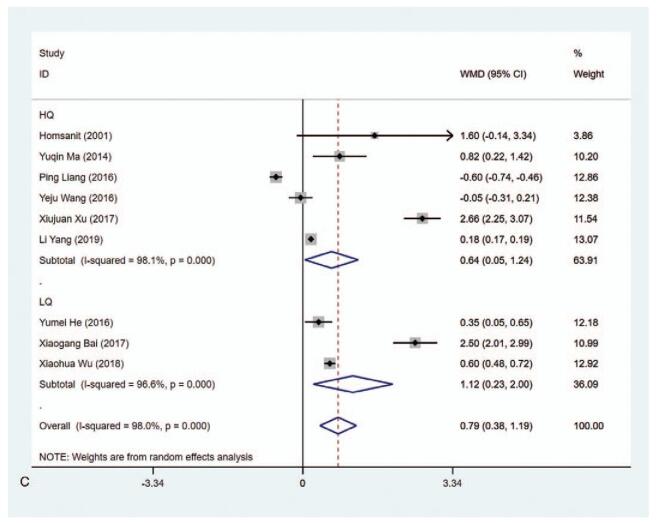
Forest plot for TSH level (A), length of study (B) and literature quality (C).

#### 3.4.4 FT_3_ level (pmol/L)

Eight studies included the data about FT_3_ level (pmol/L). The pooled data indicated that the FT_3_ level in the MMI treatment group was lower than that in the PTU treatment group (WMD = −1.388, 95% CI:−2.543 to −0.233, *P* = .019) (Fig. 6 A, Table 2). The sensitivity analysis showed that WMD−1.388 (95% CI: −2.543 to −0.233). As the heterogeneity between studies was considerable (*I*^2^ = 97.7%), subgroup analysis was conducted based on length of study and literature quality. The results showed that 1 year (WMD = −1.767, 95% CI: −2.992 to −0.542, *P* = .005) and low quality (WMD = −2.311, 95% CI:−2.667 to -1.955, *P* < .001) presented statistical differences (Fig. 6 B and C, Table 2). The results of meta-regression revealed that length of study (3 vs 6 months or 3 months vs 1 year) and literature quality (high quality vs low quality) had no effect on the heterogeneity (*P* > .05). Additionally, we found significant difference of MMI and PTU in subgroup analysis in terms of Blinding of Outcome Assessment (Yes) (WMD = −2.791, 95% CI: −3.351 to −2.230, *P* < .001), illustrating that FT_3_ level in the MMI treatment group was lower than that in the PTU treatment group in literatures with no risk of bias in Blinding of Outcome Assessment according to the Cochrane Collaboration's tool for assessing risk of bias in RCTs (Supplementary Figure 4, Table 2).

**Figure 6:**
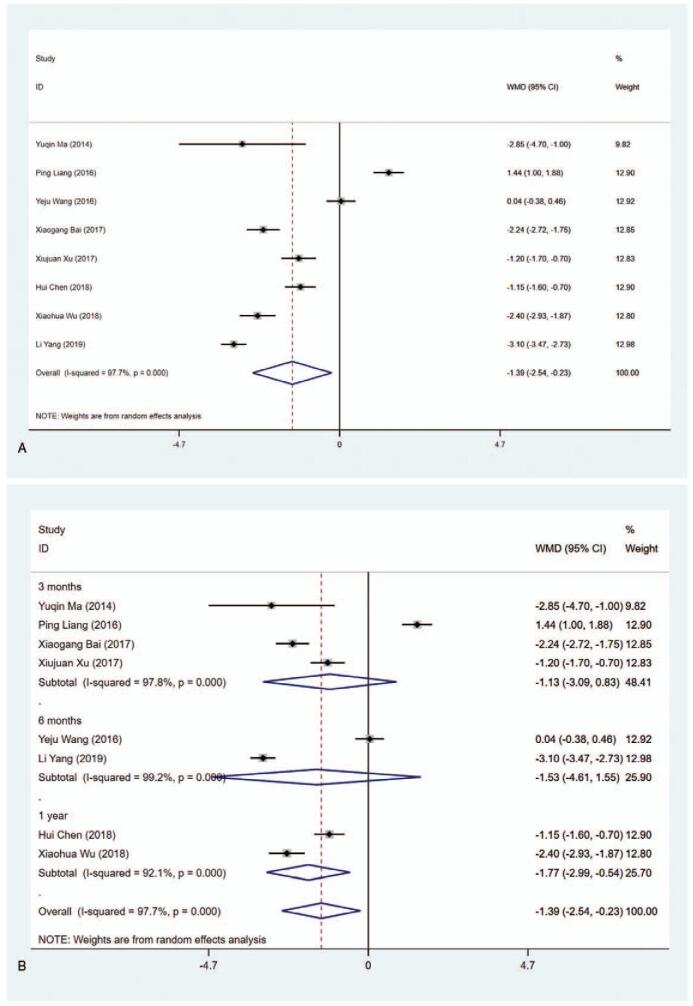
Forest plot for FT3 level (A), length of study (B) and literature quality (C).

**Figure 6 (Continued):**
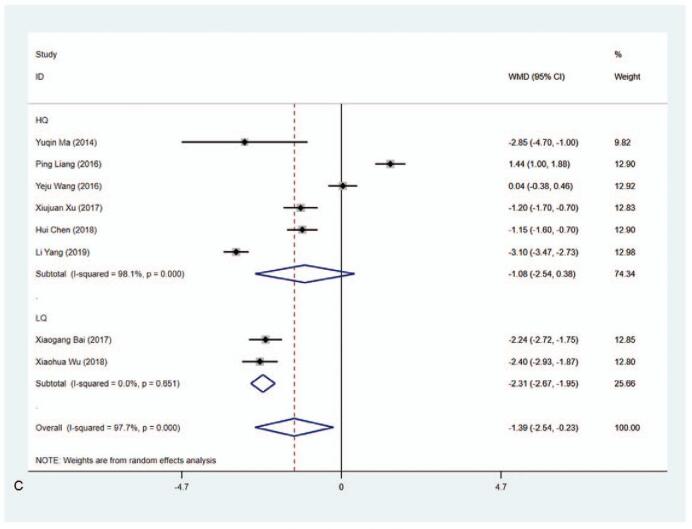
Forest plot for FT3 level (A), length of study (B) and literature quality (C).

#### 3.4.5 FT_4_ level (pmol/L)

A total of 9 articles reported the level of FT_4_ (pmol/L) and the pooled data exhibited that the level of FT_4_ was lower in the MMI treatment group than that in the PTU treatment group (WMD−3.613, 95% CI: −5.972 to −1.255, *P* = .003) (Fig. 7 A, Table 2). The sensitivity analysis showed that WMD−3.613 (95% CI: −5.972 to −1.255). The heterogeneity test results showed statistically significant difference (*I*^2^ = 98.6%). Subgroup analysis was carried out due to the substantial heterogeneity, demonstrating that there was significant difference in 1 year (WMD−4.573, 95% CI: −7.442 to −1.704, *P* = .002) (Fig. 7 B and C, Table 2). The length of study (3 vs 6 months or 3 months vs 1 year) and literature quality (high quality vs low quality) were not the sources of the heterogeneity according to the results from meta-regression. Subgroup analysis concerning the risk of bias in Blinding of Outcome Assessment according to the Cochrane Collaboration's tool for assessing risk of bias in RCTs was also performed to identify the level of FT_4_ in MMI and PTU treatment groups. The data delineated that in studies in Blinding of Outcome Assessment (No) group, the level of FT_4_ was lower in the MMI treatment group than that in the PTU treatment group (WMD = −6.759, 95% CI: −7.448 to −6.071, *P* < .001) (Supplementary Figure 5, Table 2).

**Figure 7:**
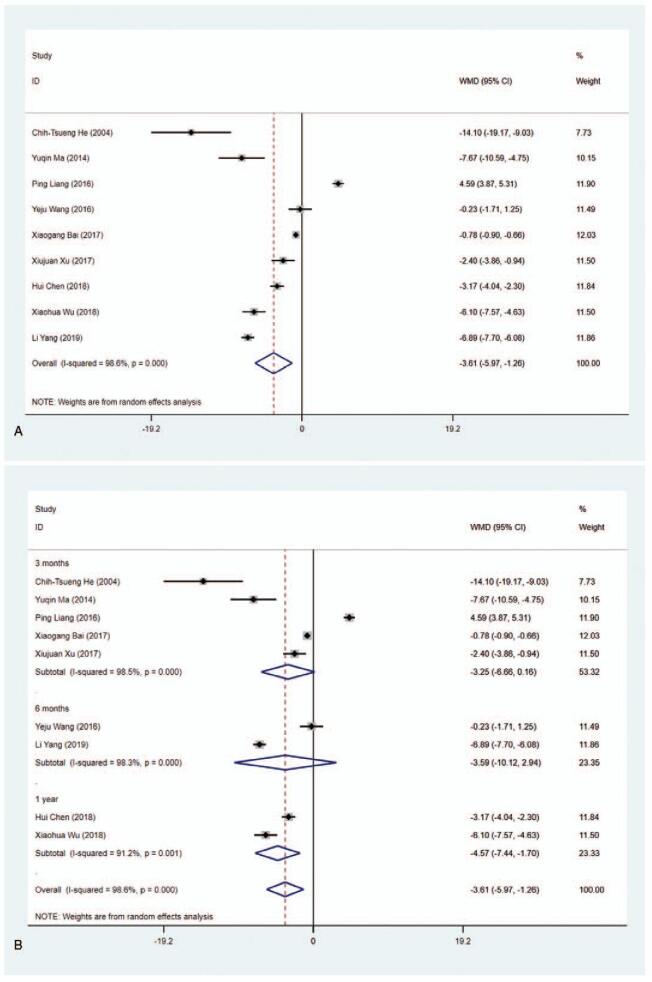
Forest plot for FT4 level (A), length of study (B) and literature quality (C).

**Figure 7 (Continued):**
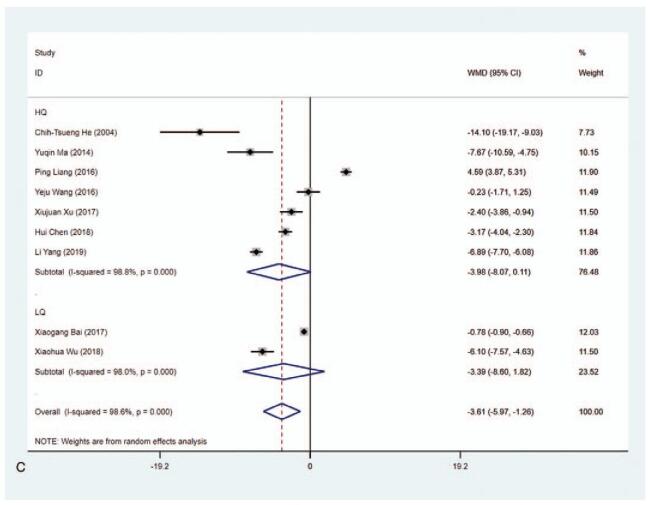
Forest plot for FT4 level (A), length of study (B) and literature quality (C).

#### 3.4.6 TRAb level

**Figure 8:**
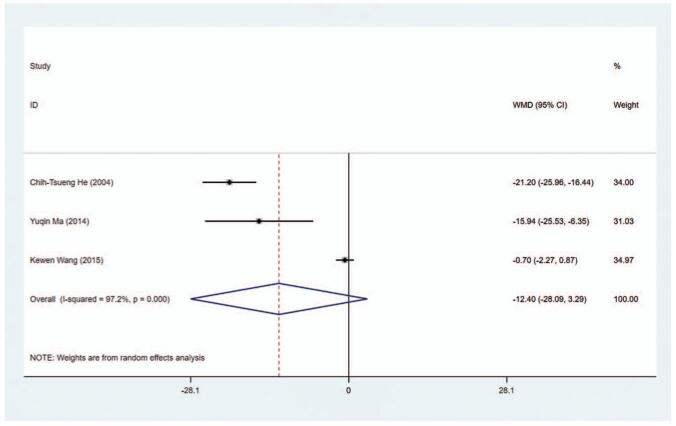
Forest plot for TRAb level.

TRAb level (U/L) as an outcome index was detected in 3 studies (*I*^2^ = 97.2%). The WMD of the pooled data in all studies was -12.398 (95% CI: −28.085 to −3.288, *P* = .121), indicating there was no statistical significance on TRAb level between the MMI treatment group and the PTU treatment group (Fig. 8, Table 2). The sensitivity analysis showed that WMD = −12.398 (95% CI: −28.085 to −3.288).

#### 3.4.7 TPOAb level

**Figure 9:**
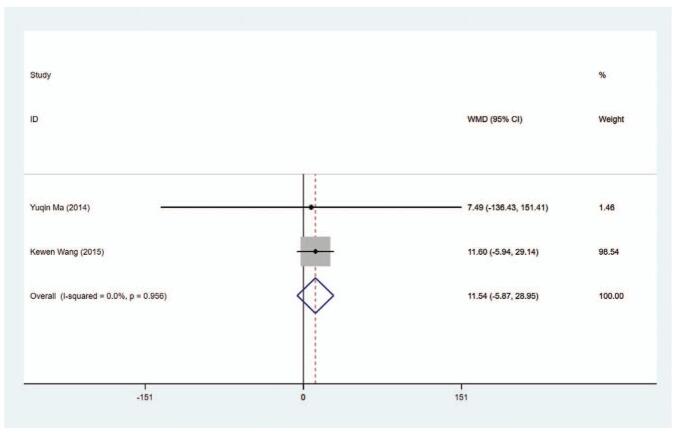
Forest plot for TPOAb level.

Totally, 2 experiments provided information about TRAb level (IU/mL) in patients. The results of heterogeneity test showed no statistically significant difference (*I*^2^ = 0.0%), so fixed-effect model was used for pooled data analysis. The results of pooled data showed that the TPOAb level had no significant difference in between the MMI treatment group and the PTU treatment group (WMD = 11.540, 95% CI: −5.873 to −28.952, *P* = .194) (Fig. 9, Table 2). The sensitivity analysis showed that WMD = 11.540 (95% CI: −5.873 to −28.952).

### 3.5 Liver function indexes

#### 3.5.1 ALP level

**Figure 10:**
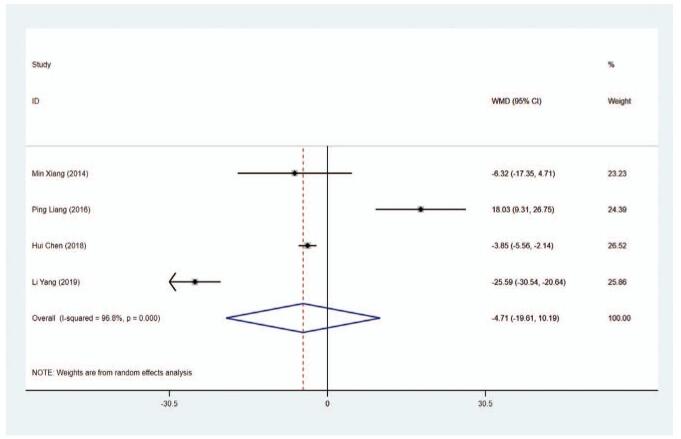
Forest plot for ALP level.

ALP level (U/L) was noticed in 4 trials. The results of the pooled data delineated that the ALP level was similar in the MMI treatment group and PTU treatment group (WMD = −4.708, 95% CI: −19.606 to −10.189, *P* = .536) (Fig. 10, Table 2). The sensitivity analysis showed that (WMD = −4.708, 95% CI: −19.606 to −10.189). To investigate the source of heterogeneity (*I*^2^ = 96.8%), meta-regression was performed on length of study, and the results indicated that length of study had no association with the heterogeneity (*P* > .05).

#### 3.5.2 ALT level

**Figure 11:**
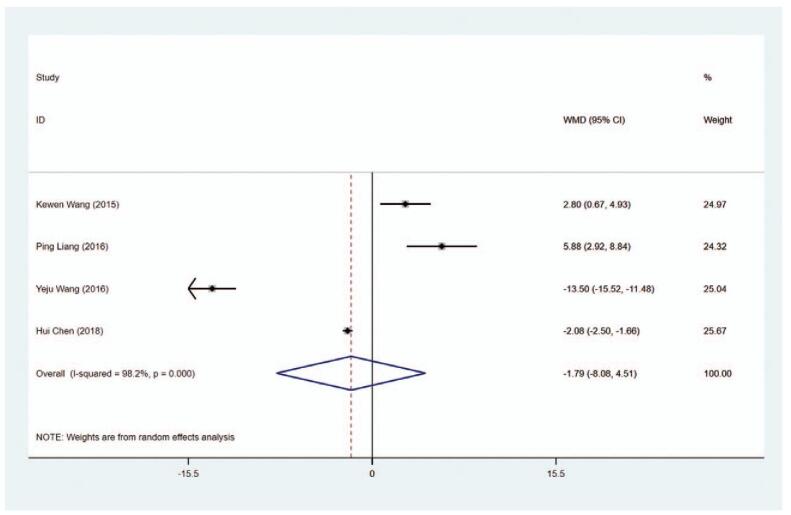
Forest plot for ALT level.

Four articles collected the data on ALT level (U/L) in patients. The WMD of the pooled data was −1.786 (95% CI: −8.078 to −4.506, *P* = .578), demonstrating the ALT level exhibited no significant difference in the MMI group and the PTU group (Figure 11, Table 2). The sensitivity analysis showed that WMD = −1.786 (95% CI: −8.078 to −4.506).

#### 3.5.3 AST level

**Figure 12:**
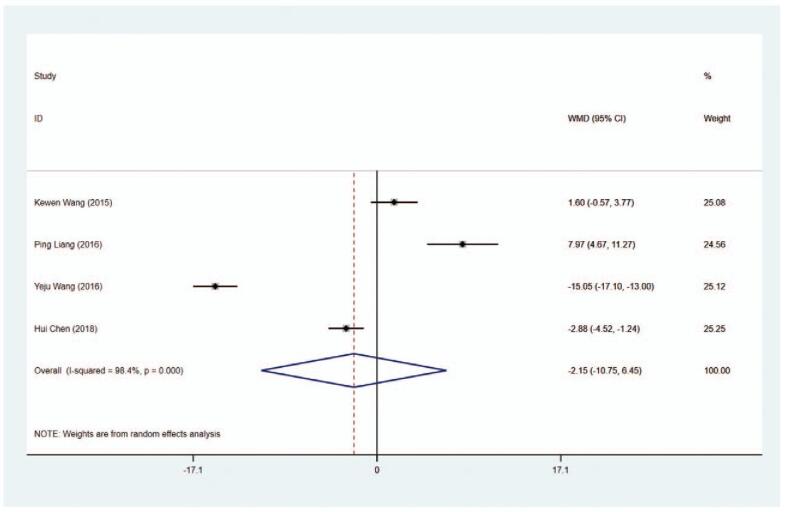
Forest plot for AST level.

Data concerning AST level (U/L) were obtained from 4 studies. As shown in Figure 12 and Table 2, no difference was obtained in AST levels between the MMI treatment group and the PTU treatment group (WMD = −2.149, 95% CI: −10.750 to −6.453, *P* = .624). The sensitivity analysis showed that WMD–2.149, (95% CI: −10.750 to −6.453).

### 3.6 Adverse reactions

#### 3.6.1 Hypothyroidism

**Figure 13:**
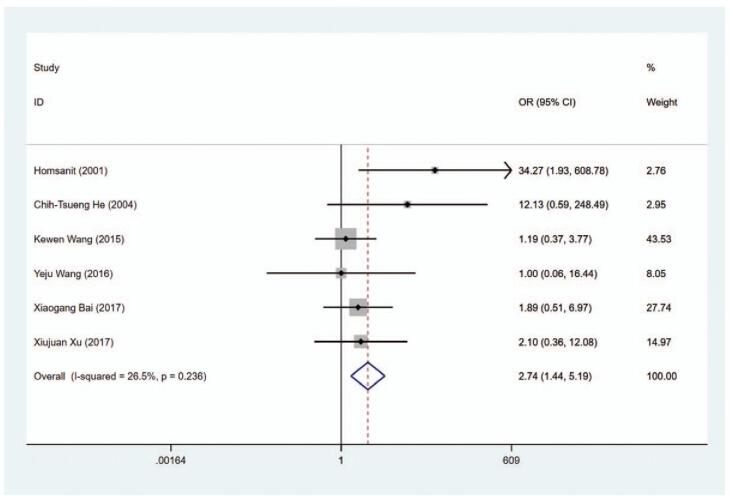
Forest plot for the risk of hypothyroidism.

The risk of hypothyroidism was analyzed in 6 trials and the results indicated that the risk of hypothyroidism was higher in the MMI treatment group than in the PTU treatment group (OR = 2.738, 95% CI 1.444–5.193, *P* = .002) (Fig. 13, Table 2). The sensitivity analysis showed that OR = 2.738 (95% CI: 1.444–5.193).

#### 3.6.2 Liver function damage

**Figure 14:**
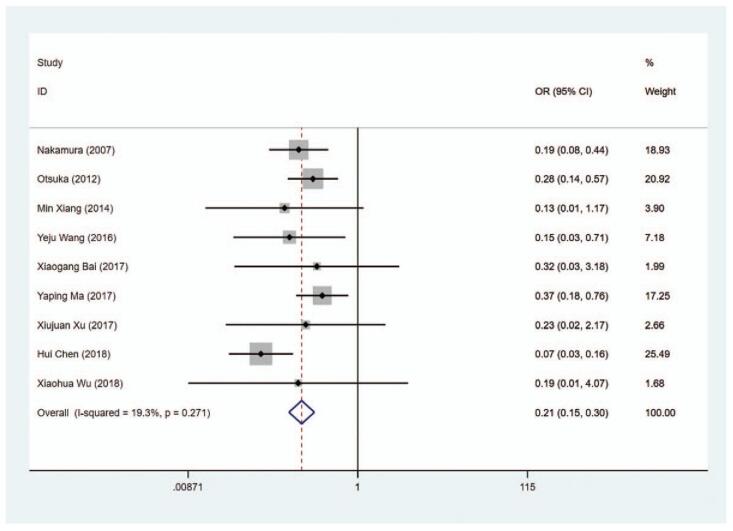
Forest plot for the risk of liver function damage.

The definition of liver function damage refers to when AST and ALT more than double the upper limit of the reference range.^[37]^ The data on liver function damage were extracted from 9 studies. We observed that the risk of liver function damage in the MMI treatment group was lower than that in the PTU treatment group (OR = 0.208, 95% CI: 0.146–0.296, *P* < .001) (Fig. 14, Table 2). The sensitivity analysis showed that OR = 0.208 (95% CI: 0.146–0.296).

#### 3.6.3 Rash

**Figure 15:**
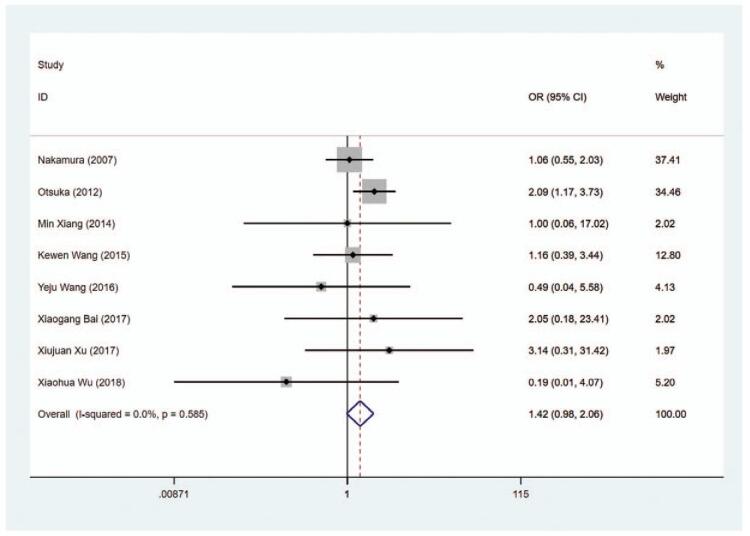
Forest plot for the risk of rash.

A total of 8 articles included the data about rash in the patients. The pooled data revealed that there was no significant difference regarding the risk of rash in the MMI treatment group and the PTU treatment group (OR = 1.419, 95% CI: 0.980–2.056, *P* = .064) (Fig. 15, Table 2). The sensitivity analysis showed that OR = 1.419 (95% CI: 0.980–2.056).

#### 3.6.4 Pruritus

**Figure 16:**
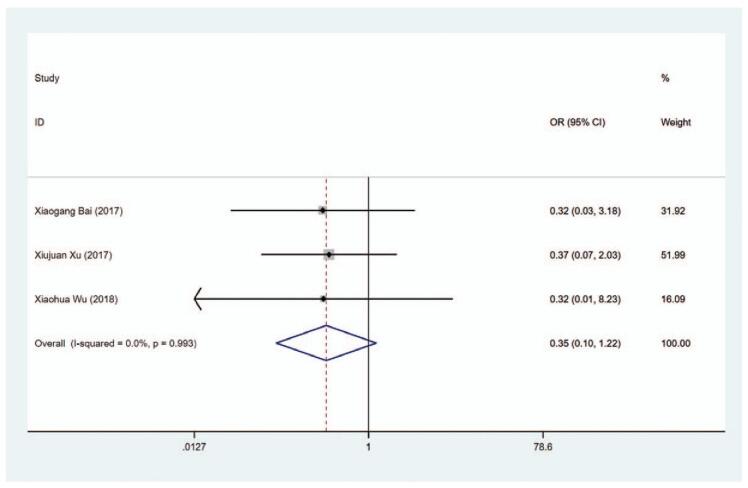
Forest plot for the risk of pruritus.

The data on the risk of pruritus in patients were available in 3 trials. As displayed in Figure 16 and Table 2, no significant difference was shown in the risk of pruritus between the MMI treatment group and the PTU treatment group (OR = 0.247, 95% CI: 0.099–1.220, *P* = .099). The sensitivity analysis showed that OR = 0.247 (95% CI: 0.099–1.220).

#### 3.6.5 Leukocytopenia

**Figure 17:**
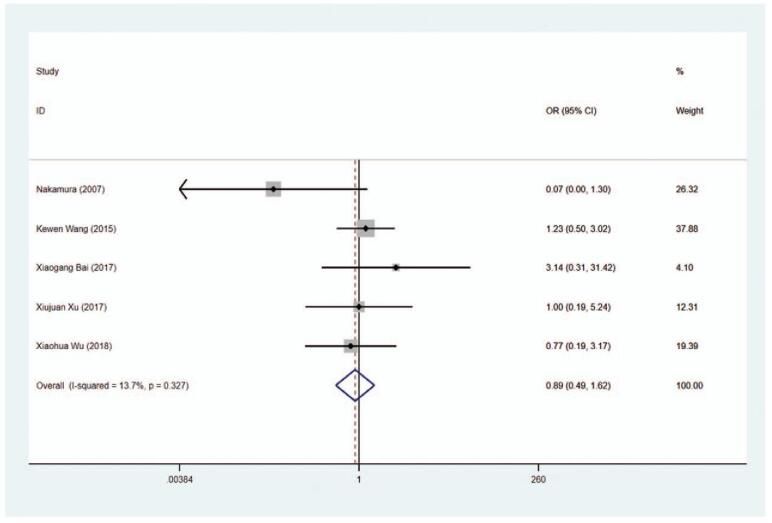
Forest plot for the risk of leukocytopenia.

A total of 5 studies analyzing the risk of leukocytopenia were included. The pooled data indicated that the risk of leukocytopenia was similar in the MMI treatment group and the PTU treatment group (OR = 0.887, 95% CI: 0.487–1.615, *P* = .696) (Fig. 17, Table 2). The sensitivity analysis showed that OR = 0.887 (95% CI: 0.487–1.615).

### 3.7 Recurrence of hyperthyroidism

**Figure 18:**
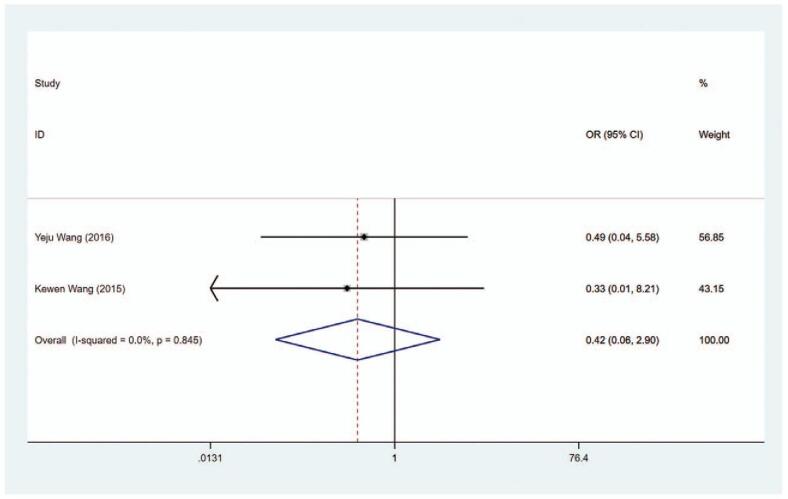
Forest plot for the recurrence of hyperthyroidism.

In total, 2 articles explored the recurrence of hyperthyroidism. The pooled data depicted that the risk of recurrence of hyperthyroidism was comparable in the MMI treatment group and the PTU treatment group (OR = 0.420, 95% CI: 0.061–2.904, *P* = .379) (Fig. 18, Table 2). The sensitivity analysis showed that OR = 0.420 (95% CI: 0.061–2.904).

## 4 Discussion

This meta-analysis compared the efficacy and safety of MMI and PTU in the treatment of hyperthyroidism. The results showed that the levels of T_3_, T_4_, FT_3_, FT_4_ and the risk of liver function damage in the MMI treatment group were lower than those in the PTU treatment group. The TSH level and the risk of hypothyroidism were higher in the MMI treatment group than those in the PTU treatment group. The findings of our study might offer a reference for the treatment of hyperthyroidism regarding ATDs.

T_3_ and T_4_ are members of iodine-containing tyrosine, 90% of them can bind to plasma proteins composed of thyroxin-binding globulin when released to blood, and only a few of them are in free state, becoming FT_3_ and FT_4_.^[38]^ The increase of T_3_ and T_4_ will inhibit the secretion of TSH. TSH serves as the first line indicator for evaluating thyroid function and the best index for screening overt and subclinical hyperthyroidism.^[39]^ MMI suppresses the peroxidase system in thyroid cells to inhibit the iodization of tyrosine which can decrease the expression of T_3_, T_4_ and increase the expression of TSH; PTU inhibits the process of transformation of T_4_ into T_3_ and further elevates the level of TSH.^[40]^ In our study, the levels of T_3_, T_4_, FT_3_ and FT_4_ in the MMI treatment group were lower than those in the PTU treatment group, whereas the level of TSH level was higher in the MMI treatment group than those in the PTU treatment group. This indicates that MMI is superior to PTU in the treatment of hyperthyroidism and can more effectively reduce the synthesis of T_3_ and T_4_. This conclusion was supported by a study from He et al indicating that MMI treatment induced a more rapid decrease of serum T_3_ levels than PTU treated patients.^[21]^ Okamura et al emphasized that MMI treatment had better effect on reducing the level T_3_ in serum than PTU treatment.^[37]^ That maybe because MMI had better effect on the substrate for T_3_ manufacture from T_4_. Heterogeneities existed in the results of T_3_, T_4_, TSH, FT_3_, and FT_4_ levels and subgroup analysis and sensitive analysis were conducted. The data depicted that significant differences were observed in 3 months, ≥1 year, high quality and low quality in T_3_ level, 3 months, high quality and low quality in T_4_ levels, ≥1 year, high quality and low quality in TSH level, 1 year and low quality in FT_3_ level and 1 year in FT_4_ level. However, meta-regression indicated the sources of the heterogeneity were not because of the length of study (3 vs 6 months or 3 months vs 1 year) and literature quality (high quality vs low quality). Additionally, based on the results of the Cochrane Collaboration's tool for assessing risk of bias in RCTs ^[19]^, subgroup analysis was also conducted based on the results of Blinding of Outcome Assessment. The data indicated that the evident differences were shown in T_3_ and T_4_ levels in Blinding of Outcome Assessment (Yes) and Blinding of Outcome Assessment (No). Statistical differences were also found in FT_3_ level in Blinding of Outcome Assessment (Yes) group. Besides, in Blinding of Outcome Assessment (No) group, the levels of TSH and FT_4_ were also significantly different between MMI and PTU groups. The reason of this may be due to Blinding of Outcome Assessment is only one of the items of the Cochrane Collaboration's tool for assessing risk of bias in RCTs.

In our study, we found the risk of liver function damage in the MMI treatment group were lower than those in the PTU treatment group. Liver function damage is a pivotal adverse event of PTU and MMI treatment in hyperthyroidism patients.^[41]^ PTU may have higher risk of liver function damage than MMI. A study from Liaw et al reported that subclinical and asymptomatic liver injury can be commonly induced by PTU.^[42]^ Tamagno revealed that PTU treatment has a higher risk of hepatotoxicity than MMI.^[43]^ According to the results from the report of Russo et al, PTU ranked the third leading cause of drug-induced liver failure requiring transplants with 23 cases receiving liver transplants between 1990 and 2007 in the United States.^[44]^ This may be because PTU can lead to active metabolites, resulting in the injury of the hepatocellular and the increase of ALT in serum. Accordingly, regular measurement of the liver function for hyperthyroidism patients undergoing PTU treatment is of great value and effective measures should be taken in time when transaminase or bilirubin rise obviously. The risk of hypothyroidism was higher in the MMI treatment group than those in the PTU treatment group in our meta-analysis. In previous study, 10 mg daily administration of MMI was found to cause spontaneous hypothyroidism in 2 patients with diffuse goiter among 36 participates.^[45]^ These findings implied that the clinicians might be careful with the dose of MMI in patients to avoid hypothyroidism.

The implication of the present study was that we identified MMI might be superior to PTU in terms of reducing T_3_, T_4_, FT_3_, and FT_4_ levels, decreasing the risk of liver function damage and increasing the level of TSH. However, some limitations existed in this study. First, this study lacked the detailed analysis on sex differences in all patients as hyperthyroidism was reported to have higher incidence in females. Secondly, the functions of MMI and PTU vary dose-dependently. The doses of MMI and PTU in all the studies were not completely unification. Thirdly, publish bias was presented in the present study because the positive results were published more easily than negative results. Besides, in the clinic, more drugs will emerge for treating hyperthyroidism and the efficacy and safety of these drugs might be analyzed by network meta-analysis to identify the best drugs for treating patients with hyperthyroidism. These limitations implied that the results of our study should be interpreted with caution.

## 5 Conclusions

This meta-analysis compared the efficacy and safety of MMI and PTU in treating hyperthyroidism. The results of it indicated that the efficacy and safety of MMI was better than PTU in patients with hyperthyroidism regarding reducing T3, T4, FT3, and FT4 levels, decreasing the risk of liver function damage and increasing the level of TSH. The findings of the present study might serve as a guide for clinicians in the treatment of hyperthyroidism.

## Acknowledgments

The authors thank the participants included in our study for their contributions.

## Author contributions

All authors participated in conceiving this study. ST and LC wrote of the manuscript. Data assessment and extraction were completed by ST, LC and LJ. LC, LJ, and XF were contributed to data analysis. ST and XF critically reviewed and edited the manuscript. All authors read and approved the final manuscript.

**Conceptualization:** Shuang Tan.

**Data curation:** Long Chen, Likun Jin, Xiaomin Fu.

**Formal analysis:** Long Chen, Likun Jin, Xiaomin Fu.

**Writing – original draft:** Shuang Tan.

**Writing – review & editing:** Shuang Tan, Long Chen, Likun Jin, Xiaomin Fu.

## Supplementary Material

Supplemental Digital Content

## Supplementary Material

Supplemental Digital Content
