# Supplementary material for: The efficiency and safety of methimazole and propylthiouracil in hyperthyroidism: A meta-analysis of randomized controlled trials
Source: Medicine (Baltimore). 2021 Jul 30;100(30):e26707. doi: 10.1097/MD.0000000000026707 (PMC8322508; doi:10.1097/MD.0000000000026707)
Supplement: Supplemental Digital Content [file medi-100-e26707-s001.doc]

Supplementary Table 1 The modified Jadad scale

| Items | Score Standard | | | Score |
| --- | --- | --- | --- | --- |
|  | 0 | 1 | 2 |  |
| Randomization | Not randomized or inappropriate method of randomization | The study was described as randomized | The method of randomization was described and it was appropriate |  |
| Concealment of allocation | Not describe the method of allocation concealment | The study was described as using allocation concealment method | The method of allocation concealment was described appropriately |  |
| Double blinding | No blind or inappropriate method of blinding | The study was described as doubled blind | The method of double blinding was described and it was appropriate |  |
| Withdrawals and dropouts | Not describe the follow-up | A description of withdrawals and dropouts |  |  |
| Total | | | |  |

Supplementary Table 2 Quality evaluation results via the modified Jadad scale

| Author | Year | Randomization | Concealment of allocation | Double blinding | Withdrawals and dropouts | Total |
| --- | --- | --- | --- | --- | --- | --- |
| Homsanit | 2001 | 1 | 2 | 1 | 1 | 5 |
| Chih-Tsueng He | 2004 | 1 | 2 | 1 | 0 | 4 |
| Nakamura | 2007 | 2 | 2 | 1 | 1 | 6 |
| Otsuka | 2012 | 1 | 2 | 1 | 0 | 4 |
| Yuqin Ma | 2014 | 1 | 2 | 0 | 1 | 4 |
| Min Xiang | 2014 | 2 | 1 | 0 | 1 | 4 |
| Kewen Wang | 2015 | 1 | 1 | 1 | 0 | 3 |
| Yumei He | 2016 | 2 | 0 | 0 | 0 | 2 |
| Ping Liang | 2016 | 1 | 2 | 1 | 1 | 5 |
| Yeju Wang | 2016 | 1 | 2 | 1 | 0 | 4 |
| Xiaogang Bai | 2017 | 1 | 0 | 1 | 0 | 2 |
| Yaping Ma | 2017 | 1 | 1 | 0 | 1 | 3 |
| Xiujuan Xu | 2017 | 2 | 1 | 1 | 1 | 5 |
| Hui Chen | 2018 | 1 | 2 | 0 | 1 | 4 |
| Xiaohua Wu | 2018 | 1 | 1 | 0 | 1 | 3 |
| Li Yang | 2019 | 2 | 2 | 0 | 0 | 4 |

Supplementary Table 3 Cochrane risk of bias evaluation

| Author | Year | Random Sequence Generation | Allocation Concealment | Blinding of Participants and Personnel | Blinding of Outcome Assessment | Incomplete Outcome Data Addressed | Free of Selective Reporting | Free of Other Bias |
| --- | --- | --- | --- | --- | --- | --- | --- | --- |
| Homsanit | 2001 | Yes | Yes | Yes | Yes | Yes | Yes | Yes |
| Chih-Tsueng He | 2004 | Yes | Yes | Yes | Yes | Yes | Yes | Yes |
| Nakamura | 2007 | Yes | Yes | Yes | Yes | Yes | Yes | Yes |
| Otsuka | 2012 | Yes | Yes | Yes | Yes | Yes | Yes | Yes |
| Yuqin Ma | 2014 | Yes | Yes | Yes | No | Yes | Yes | Yes |
| Min Xiang | 2014 | Yes | Yes | Yes | No | Yes | Yes | Yes |
| Kewen Wang | 2015 | Yes | Yes | Yes | Yes | Yes | Yes | Yes |
| Yumei He | 2016 | Yes | Yes | Yes | No | ？ | Yes | Yes |
| Ping Liang | 2016 | Yes | Yes | Yes | Yes | Yes | Yes | Yes |
| Yeju Wang | 2016 | Yes | Yes | Yes | Yes | Yes | Yes | Yes |
| Xiaogang Bai | 2017 | Yes | Yes | Yes | Yes | ？ | Yes | Yes |
| Yaping Ma | 2017 | Yes | Yes | Yes | No | Yes | Yes | Yes |
| Xiujuan Xu | 2017 | Yes | Yes | Yes | Yes | Yes | Yes | Yes |
| Hui Chen | 2018 | Yes | Yes | Yes | Yes | Yes | Yes | Yes |
| Xiaohua Wu | 2018 | Yes | Yes | Yes | No | Yes | Yes | Yes |
| Li Yang | 2019 | Yes | Yes | Yes | No | Yes | Yes | Yes |

Yes=low risk of bias; No=high risk of bias;？=unclear risk of bias.

Supplementary Table 4 Results of GRADE approach for evaluating the quality of evidence

| Study | Study design | Decrease quality of evidence | | | | | Increase quality of evidence | | | Grade |
| --- | --- | --- | --- | --- | --- | --- | --- | --- | --- | --- |
|  |  | Study limitation | Indirectness | Inconsistency | Imprecision | Publication bias | Large magnitude of effect | Residual confounding | Dose-response gradient |  |
| Homsanit 2001 | RCT | 0 | 0 | 0 | 0 | 0 | 0 | 0 | 0 | High |
| Chih-Tsueng He 2004 | RCT | 0 | 0 | 0 | 0 | 0 | 0 | 0 | 0 | High |
| Nakamura 2007 | RCT | 0 | 0 | 0 | 0 | 0 | 0 | 0 | 0 | High |
| Otsuka 2012 | RCT | 0 | 0 | 0 | 0 | 0 | 0 | 0 | 0 | High |
| Yuqin Ma 2014 | RCT | 0 | 0 | 0 | 0 | 0 | 0 | 0 | 0 | High |
| Min Xiang 2014 | RCT | 0 | 0 | 0 | 0 | 0 | 0 | 0 | 0 | High |
| Kewen Wang 2015 | RCT | 0 | 0 | 0 | 0 | 0 | 0 | 0 | 0 | High |
| Yumei He 2016 | RCT | -2 | 0 | 0 | -1 | 0 | 0 | 0 | 0 | Low |
| Ping Liang 2016 | RCT | 0 | 0 | -2 | 0 | 0 | 0 | +1 | 0 | Moderate |
| Yeju Wang 2016 | RCT | 0 | 0 | -1 | 0 | 0 | 0 | 0 | 0 | Moderate |
| Xiaogang Bai 2017 | RCT | -1 | 0 | 0 | -1 | 0 | 0 | 0 | 0 | Low |
| Yaping Ma 2017 | RCT | -1 | 0 | 0 | 0 | 0 | 0 | 0 | 0 | Moderate |
| Xiujuan Xu 2017 | RCT | 0 | 0 | 0 | 0 | 0 | 0 | 0 | 0 | High |
| Hui Chen 2018 | RCT | 0 | 0 | 0 | 0 | 0 | 0 | 0 | 0 | High |
| Xiaohua Wu 2018 | RCT | 0 | 0 | 0 | 0 | 0 | 0 | 0 | 0 | High |
| Li Yang 2019 | RCT | 0 | 0 | 0 | 0 | 0 | 0 | 0 | 0 | High |
