# Supplementary material for: The efficiency and safety of methimazole and propylthiouracil in hyperthyroidism: A meta-analysis of randomized controlled trials
Source: Medicine (Baltimore). 2021 Jul 30;100(30):e26707. doi: 10.1097/MD.0000000000026707 (PMC8322508; doi:10.1097/MD.0000000000026707)

Supplementary Figure 1 Forest plot for T_3_ level concerning Blinding of Outcome Assessment.


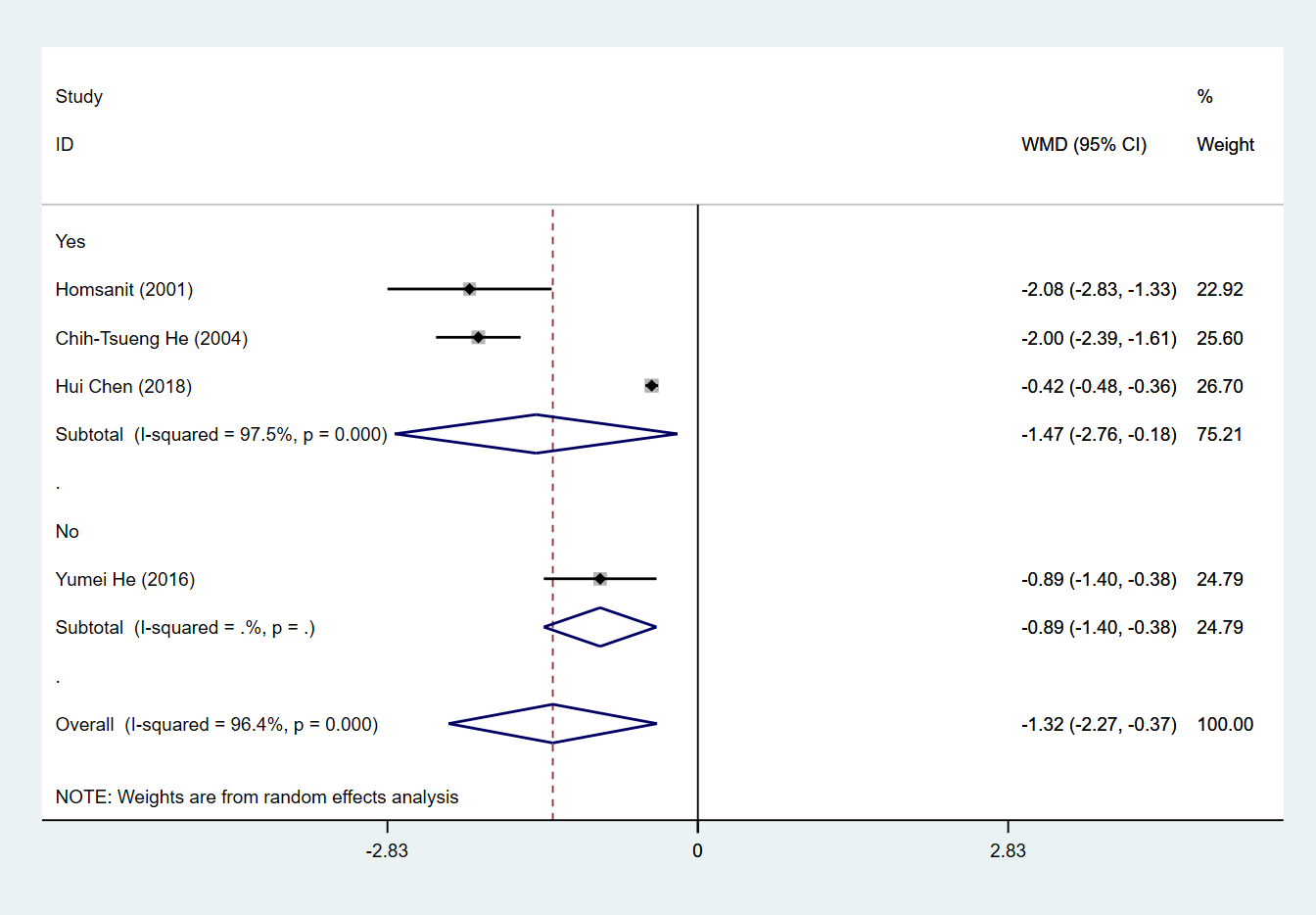


Supplementary Figure 2 Forest plot for T_4_ level concerning Blinding of Outcome Assessment.


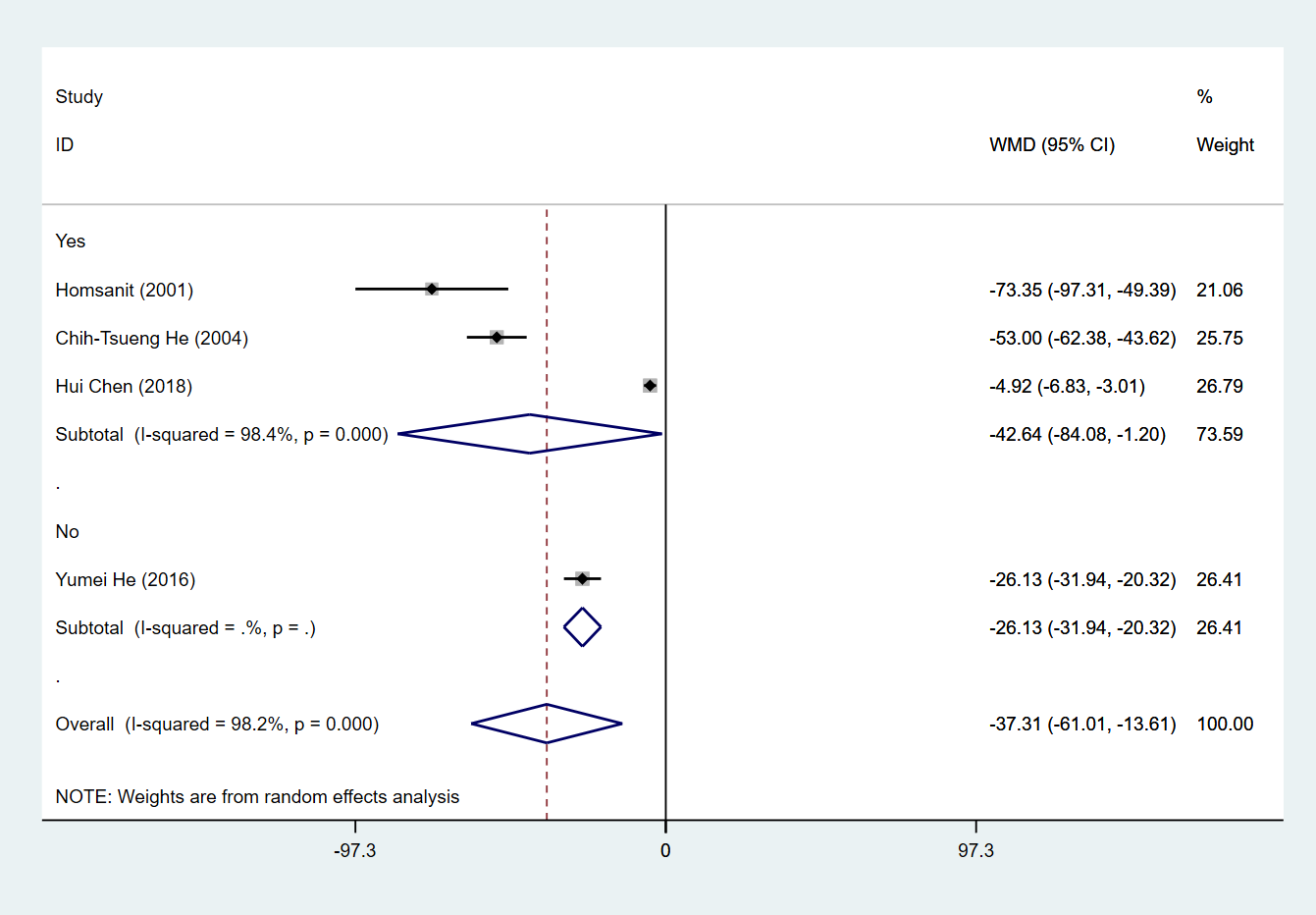


Supplementary Figure 3 Forest plot for TSH level concerning Blinding of Outcome Assessment.


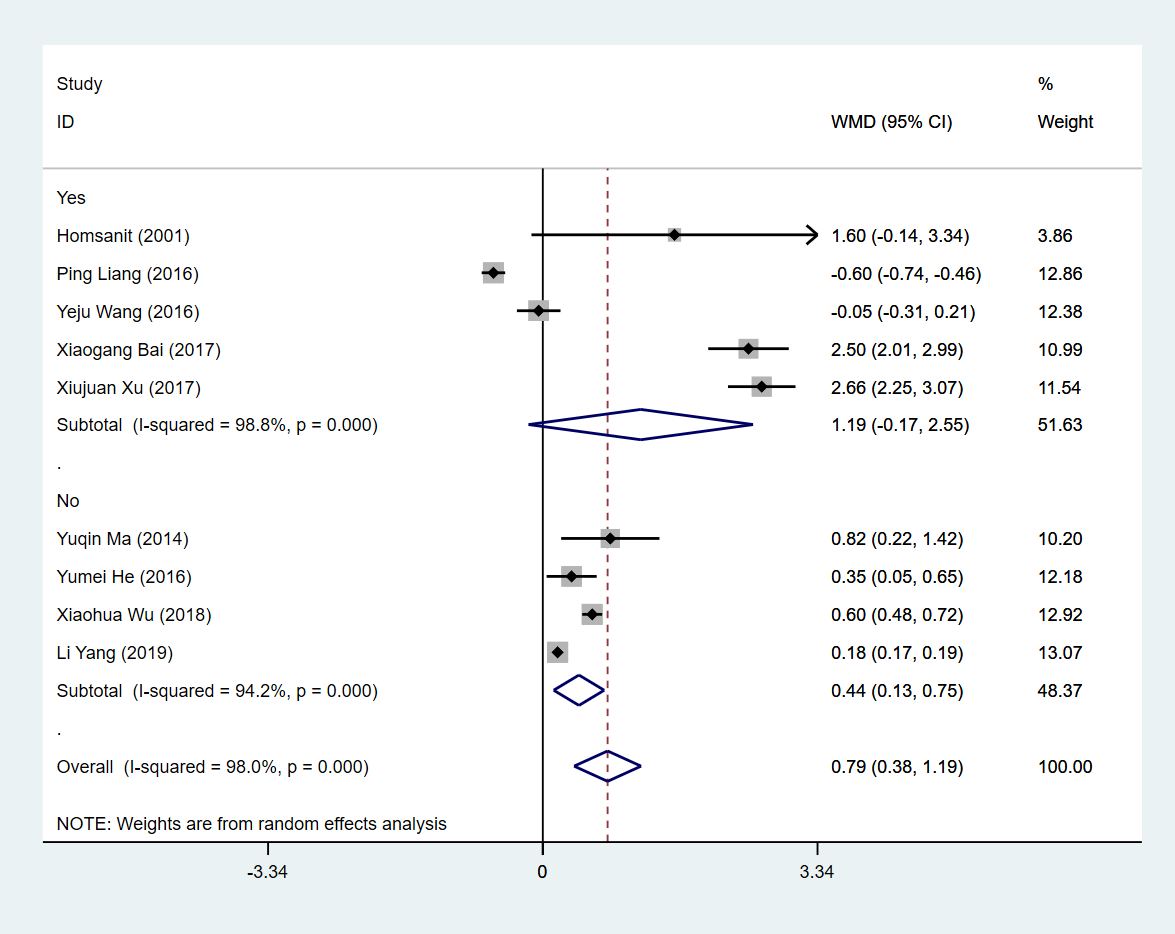


Supplementary Figure 4 Forest plot for FT_3_ level concerning Blinding of Outcome Assessment.


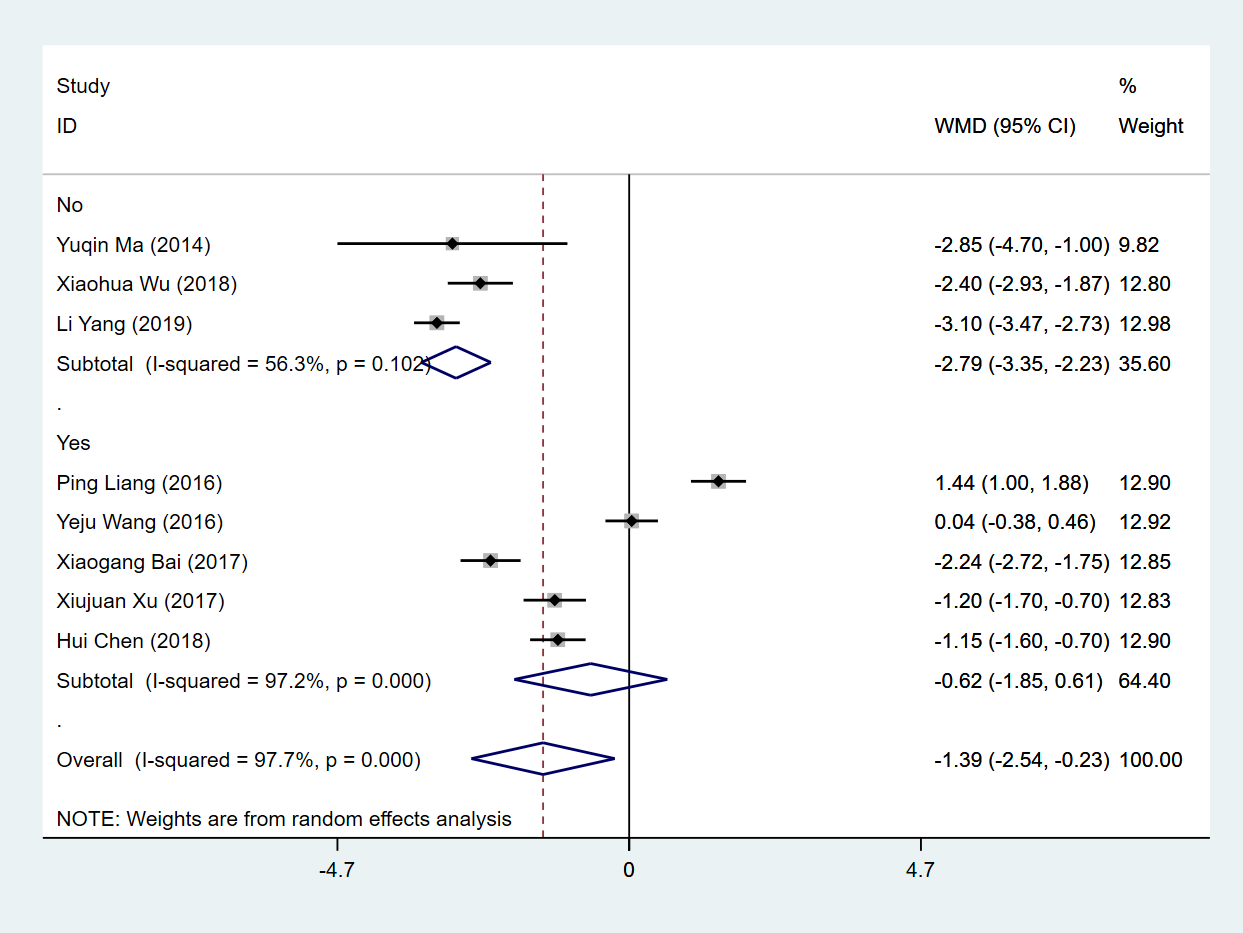


Supplementary Figure 5 Forest plot for FT_4_ level concerning Blinding of Outcome Assessment.


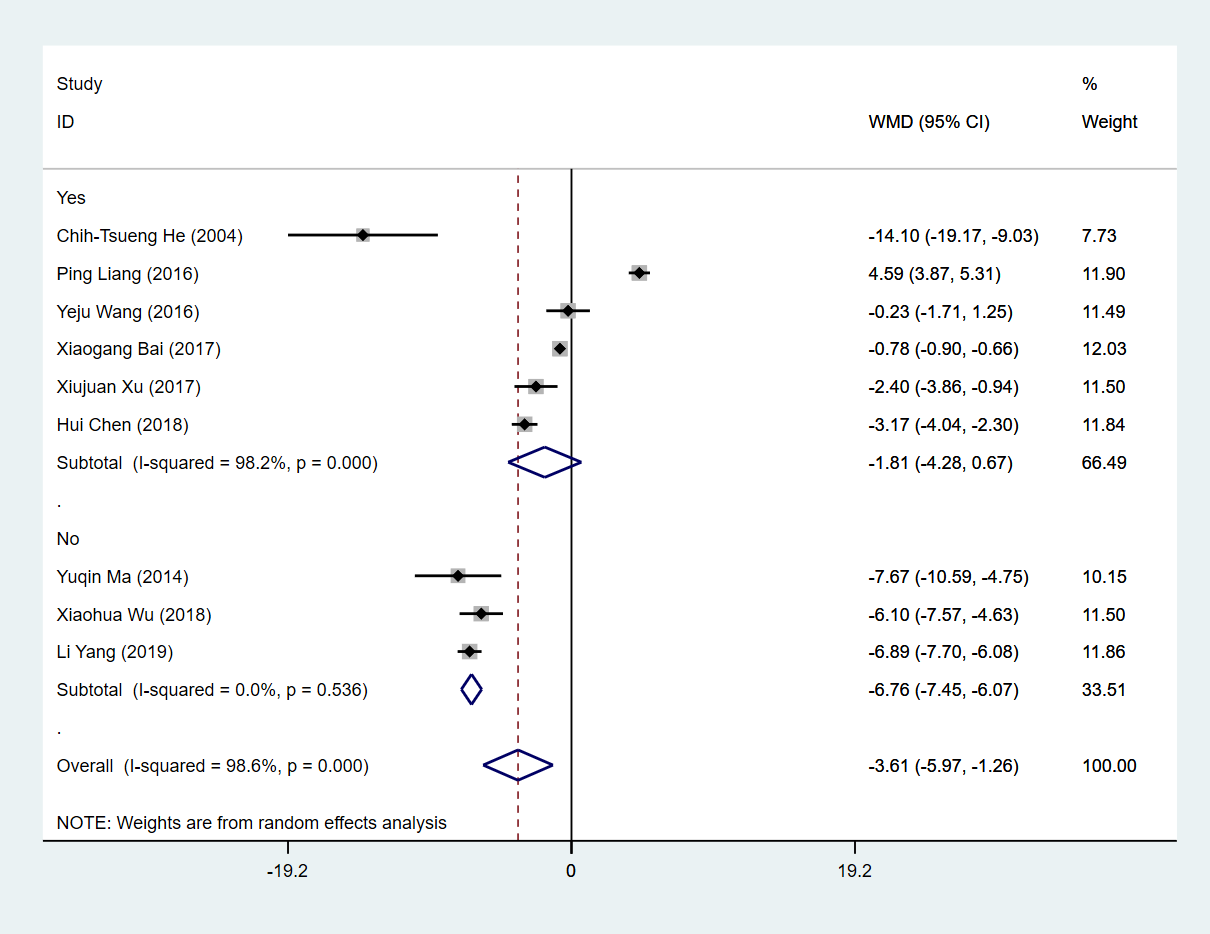


Supplementary Figure 6 The presentation of risk of bias based on Cochrane Collaboration’s tool.


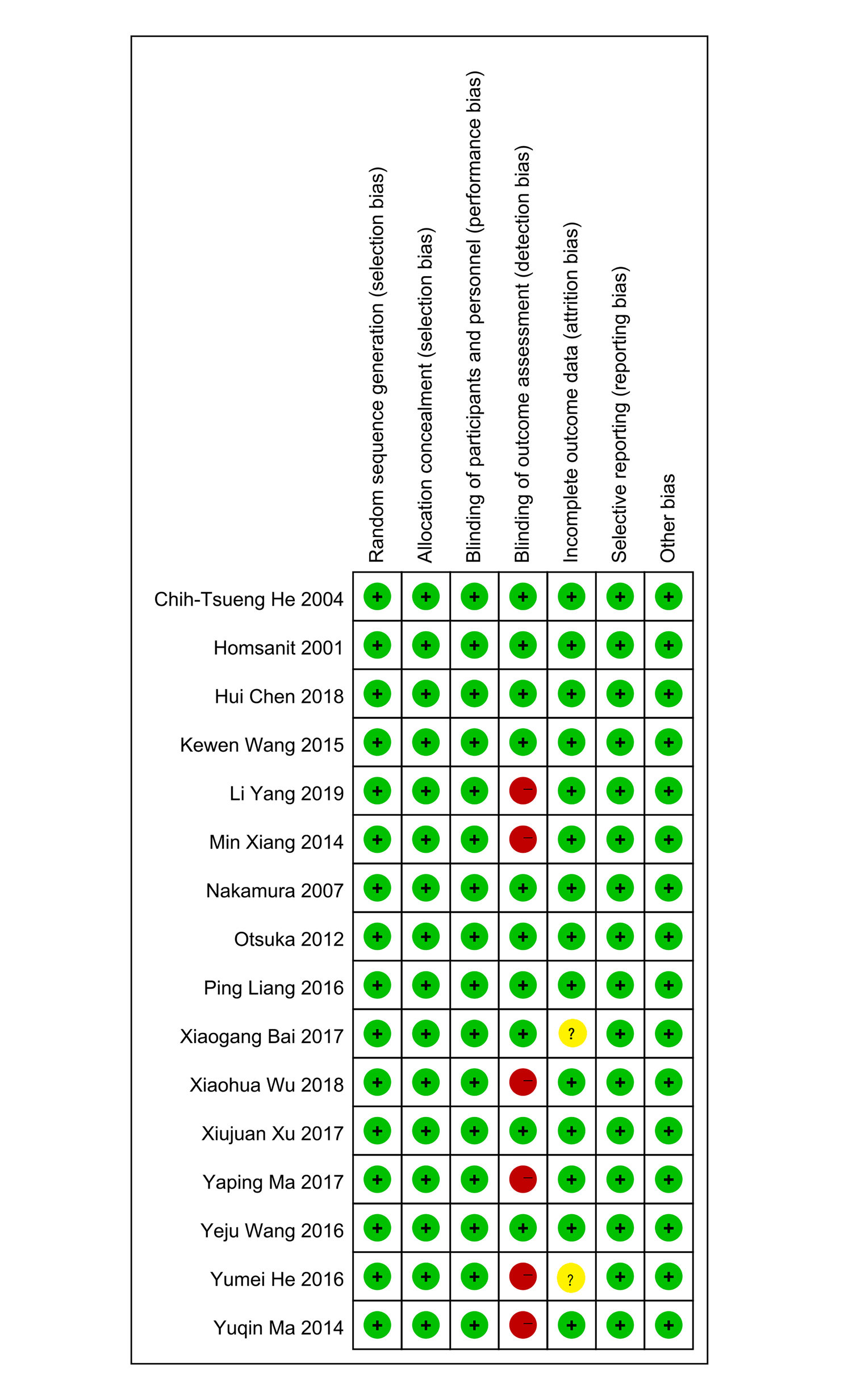

Supplement: Supplemental Digital Content [file medi-100-e26707-s002.doc]
